# Potential Enterotoxicity of Phylogenetically Diverse Bacillus cereus Sensu Lato Soil Isolates from Different Geographical Locations

**DOI:** 10.1128/AEM.03032-19

**Published:** 2020-05-19

**Authors:** Justyna Malgorzata Drewnowska, Natalia Stefanska, Magdalena Czerniecka, Grzegorz Zambrowski, Izabela Swiecicka

**Affiliations:** a Department of Microbiology, Faculty of Biology, University of Bialystok, Bialystok, Poland; b Department of Cytobiochemistry, Faculty of Biology, University of Bialystok, Bialystok, Poland; c Laboratory of Applied Microbiology, University of Bialystok, Bialystok, Poland; d Laboratory of Tissue Culture, Faculty of Biology, University of Bialystok, Bialystok, Poland; INRS—Institut Armand-Frappier

**Keywords:** *Bacillus cereus sensu lato*, genetic structure, enterotoxicity, phylogeny, ecology

## Abstract

Bacillus cereus sensu lato comprises Gram-positive spore-forming bacteria producing toxins associated with foodborne diseases. Three pore-forming enterotoxins, nonhemolytic enterotoxin (Nhe), hemolysin BL (Hbl), and cytotoxin K (CytK), are considered the primary factors in B. cereus sensu lato diarrhea. The aim of this study was to determine the potential risk of enterotoxicity among soil B. cereus sensu lato isolates representing diverse phylogroups and originated from different geographic locations with various climates (Burkina Faso, Kenya, Argentina, Kazakhstan, and Poland). While *nheA*- and *hblA*-positive isolates were present among all B. cereus sensu lato populations and distributed across all phylogenetic groups, *cytK-2*-positive strains predominated in geographic regions with an arid hot climate (Africa) and clustered together on a phylogenetic tree mainly within mesophilic groups III and IV. The highest *in vitro* cytotoxicity to Caco-2 and HeLa cells was demonstrated by the strains clustered within phylogroups II and IV. Overall, our results suggest that B. cereus sensu lato pathogenicity is a comprehensive process conditioned by many intracellular factors and diverse environmental conditions.

**IMPORTANCE** This research offers a new route for a wider understanding of the dependency between pathogenicity and phylogeny of a natural bacterial population, specifically within Bacillus cereus sensu lato, that is widely distributed around the world and easily transferred into food products. Our study indicates differences in the phylogenetic and geographical distributions of potential enterotoxigenic B. cereus sensu lato strains. Hence, these bacilli possess a risk for human health, and rapid testing methods for their identification are greatly needed. In particular, the detection of the CytK enterotoxin should be a supporting strategy for the identification of pathogenic B. cereus sensu lato.

## Abstract (precis)

This research offers a new route for a wider understanding of the dependency between pathogenicity and phylogeny of a natural bacterial population, specifically within Bacillus cereus sensu lato, that is widely distributed around the world and easily transferred into food products. Our study indicates differences in the phylogenetic and geographical distributions of potential enterotoxigenic B. cereus sensu lato strains. Hence, these bacilli possess a risk for human health, and rapid testing methods for their identification are greatly needed. In particular, the detection of the CytK enterotoxin should be a supporting strategy for the identification of pathogenic B. cereus sensu lato.

## INTRODUCTION

Bacillus cereus sensu lato comprises soil-dwelling endospore-forming bacilli with species discrimination based on their phenotypes associated mostly with genes of plasmidic origin (1, 2). B. cereus sensu stricto (here, B. cereus), an opportunistic foodborne pathogen (3, 4), B. anthracis, the etiological agent of anthrax (1), and B. thuringiensis, an entomopathogen widely used as a biopesticide (5), are the most well-known members of the group. Bacillus cytotoxicus, which is occasionally responsible for severe food poisoning (6), and B. wiedmannii, cytotoxic in a HeLa cell model (7), also have pathogenic potential. Likewise, B. cereus sensu lato includes bacteria with a seemingly irrelevant medical significance, such as Bacillus toyonensis (8), B. mycoides, and B. pseudomycoides (9, 10); however, their ability to cause diseases cannot be excluded, e.g., potentially foodborne emetic B. weihenstephanensis (presently reclassified as B. mycoides [11]) strains have been described (12). Recently, Bacillus manliponensis (13), B. gaemokensis (14), B. bombysepticus (15), B. bingmayongensis (16), B. paranthracis, B. pacificus, B. tropicus, B. albus, B. mobilis, B. luti, B. proteolyticus, B. nitratireducens, and B. paramycoides (17) were also proposed as new members of the group.

Most of studies have focused on foodborne and clinical B. cereus sensu lato isolates (4, 7, 18–21), yet strains belonging to this group are present in various natural environments, such as soil (22) or aquatic habitats (23), from which they can be easily transferred to crops and food products (24). In addition, since B. cereus sensu lato spores are heat resistant, they can survive the cooking process, presenting a serious risk of foodborne diseases (20). Likewise, a form of food intoxication called the emetic or “fried rice” syndrome caused by B. cereus (4, 25) producing a heat-stable emetic toxin, cereulide (26), has been observed. Nevertheless, gastrointestinal infections known as the diarrheal syndrome are noted most often (27) and usually result in abdominal pain and diarrhea 8 to 16 h after the ingestion of food contaminated with bacterial spores (3). Among the most common factors of B. cereus sensu lato diarrhea are tripartite enterotoxin complexes, hemolysin BL (Hbl) and nonhemolytic enterotoxin (Nhe), both belonging to the α-helical pore-forming toxin family (3). Interestingly, in 1998, a fatal food poisoning outbreak was reported in France, and it was associated with B. cereus producing a single enterotoxin named cytotoxin K (CytK), which belongs to the β-barrel pore-forming toxin family and shows both necrotic and hemolytic properties (28). Two CytK variants, CytK-1 and CytK-2, which display different pathogenicities, have been described. CytK-1 is more cytotoxic than CytK-2 on Caco-2 and Vero cells (29); thus, the isolates harboring *cytK-1* were classified as a separate species, B. cytotoxicus (6). Nevertheless, it is worth noting that other enterotoxins, phospholipases, and metalloproteases can contribute to B. cereus sensu lato pathogenesis (3, 18). Secretion of the majority of virulence factors by B. cereus sensu lato (with the exception of B. anthracis) is regulated by the pleiotropic phospholipase C regulator (PlcR), which belongs to the PlcR/PapR quorum-sensing system (30, 31). While Hbl production and Nhe production have been investigated in numerous studies (4, 6, 7, 20, 32–34), CytK synthesis and its role in diarrheal infection are still poorly understood, especially in environmental B. cereus isolates.

An extensive study on the dynamics of bacterial speciation showed that among individual bacterial species, so-called ecotypes comprising ecologically distinct bacterial populations can occur (35). In B. cereus sensu lato, seven thermal ecotypes adapted to different thermal niches were proposed (36), which correlate well with ribosomal protein profiles (37) and the group’s phylogeny (22). As Guinebretière and coworkers (38) showed that food poisoning potential appeared to correlate with phylogenetic groups and different distributions for emetic and diarrheal disease are observed between countries (25), we assumed that B. cereus sensu lato strains from an individual geographic location group together into different thermal phylogenetic groups that possess various enterotoxic potentials. Thus, the goal of this study was to determine the potential risk of enterotoxicity among soil populations representing various phylogroups and originating from different geographic locations with varied climates, including arid hot steppe (Burkina Faso, Kenya), wet savanna (Kenya), a mild climate with hot summers (Argentina), and continental cold climates with hot (Kazakhstan) and warm (Poland) summers.

## RESULTS

### Worldwide distribution of B. cereus sensu lato isolates harboring the enterotoxin determinants.

**FIG 1:**
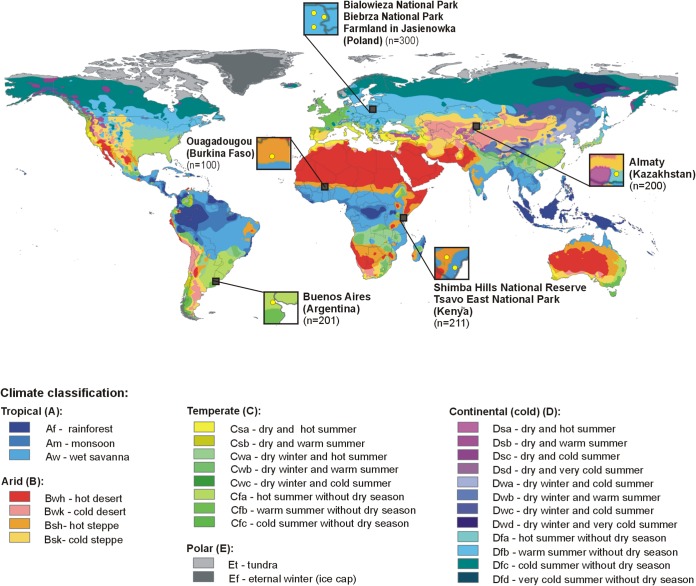
Distribution of B. cereus sensu lato soil strains with regard to the world Köppen-Geiger climate classification. Numbers of bacteria originating from individual locations are given in parentheses. Map adapted from that by Peel and coworkers (39).

**FIG 2:**
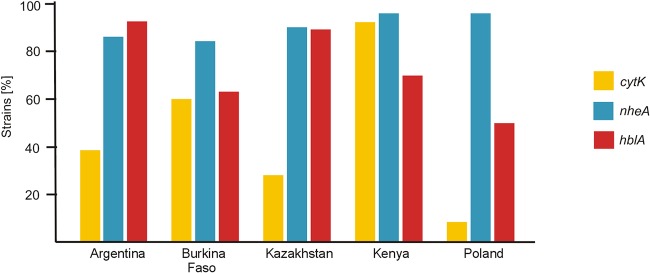
Percentages of Bacillus cereus sensu lato strains harboring enterotoxin genes.

Among 1,012 B. cereus sensu lato isolates originating from soil samples obtained from geographically different locations (Fig. 1, with a map adapted from Peel and coworkers [39]), the *cytK* gene was present in 40.6% of the isolates (see also Table S1 in the supplemental material). The c*ytK-1* variant was not found in any population, while the *cytK-2*-positive strains were noted in the highest proportion in Kenyan bacilli from Tsavo East National Park and Shimba Hills National Reserve (91.9%) as well as among Burkinabe isolates from an urban park in Ouagadougou (60.0%), followed by Argentinian strains from a soil sample taken in a park in Buenos Aires (38.3%) and Kazakh bacilli from a park in Almaty (28.0%). The lowest frequency of the *cytK-2*-positive isolates (8.0%) was observed among Polish B. cereus sensu lato strains, isolated from soil samples taken in Bialowieza National Park, Biebrza National Park, and a farm in Jasienowka (Fig. 2). The observed proportions of the *cytK-2*-positive strains differed significantly among populations originating from distinct geographic locations (with a *P* value of <0.05 in the χ^2^ test).

To have a wider view of the potential B. cereus sensu lato enterotoxicity, we also analyzed the frequency of other determinants encoding the most often noted enterotoxins, Nhe and Hbl. Among all populations, the *nheA* and *hblA* genes were present in 91.5% and 71.3% of the tested isolates, respectively (Table S1). Yet, we observed a high proportion of the *nheA*-positive strains in all populations, ranging from 84.0% in Burkinabe isolates to 95.7% in both Kenyan and Polish strains (Fig. 2), whereas the highest frequency of the *hblA*-positive isolates among these bacilli was noted in B. cereus sensu lato from Argentina (92.0%) and Kazakhstan (89.0%), followed by Kenyan (69.7%), Burkinabe (63.0%), and Polish (49.7%) strains. For the *hblA*-positive strains, the observed proportions from distinct geographic locations differed significantly from each other (with a *P* value of <0.05 in the χ^2^ test).

### Genetic structure of Bacillus cereus sensu lato populations versus *cytK-2*, *hblA*, and *nheA* gene presence.

**FIG 3:**
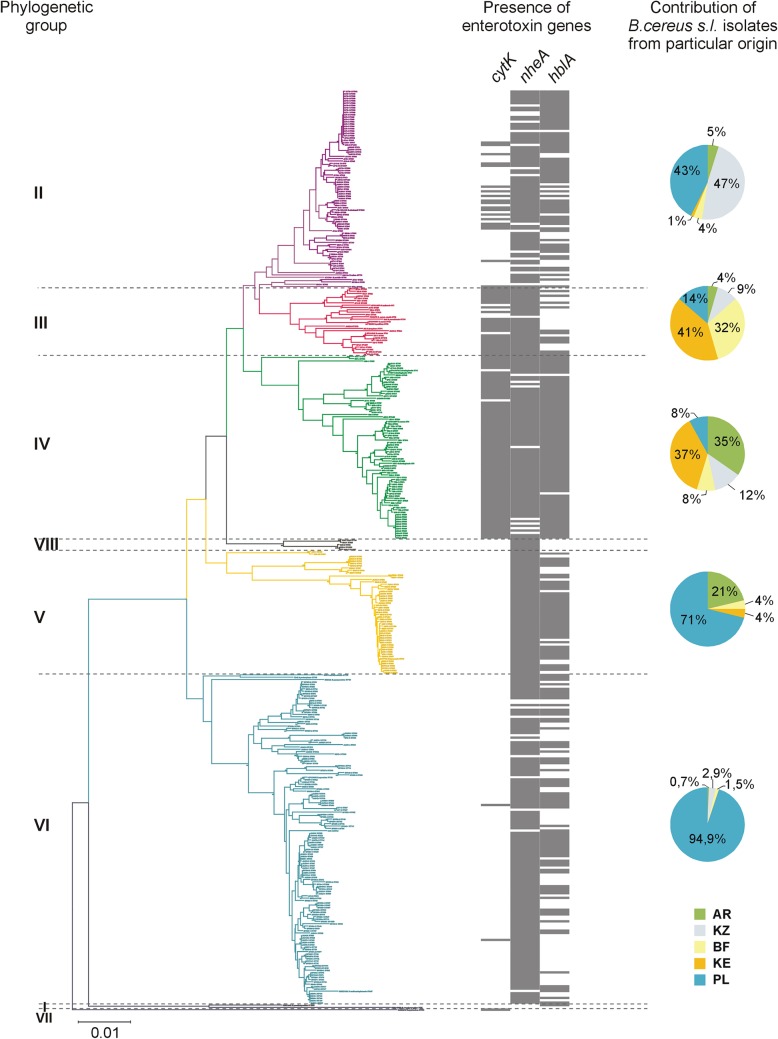
Phylogenetic relatedness of Bacillus cereus sensu lato strains harboring genetic determinants of potential enterotoxicity and the percentage contribution of B. cereus sensu lato from different origins within particular phylogenetic groups. The phylogenetic tree of 396 B. cereus sensu lato strains was constructed based on seven concatenated housekeeping loci (MLST method) representing a total of 2,829 nucleotides. The evolutionary history was deduced using the neighbor-joining (NJ) method in MEGA7 software. Branch quality was evaluated using a 1,000-replicate bootstrap test. The optimal tree was drawn to scale, with branch lengths in the same units as those of the evolutionary distances used to infer the phylogenetic tree. The evolutionary distances were computed using the maximum composite likelihood method and are in the units of the number of base substitutions per site. The presence of the corresponding gene (*cytK-2*, *nheA*, and *hblA)* is marked by gray lines next to the appropriate isolates on the phylogenetic tree. The negative results were omitted for clarity. AR, Argentina; KZ, Kazakhstan; BF, Burkina Faso; KE, Kenya; PL, Poland.

A phylogenetic study based on multilocus sequence type (MLST) enabled clustering of 372 randomly chosen environmental strains represented by 254 sequence types (STs) to five major phylogenetic groups (II to VI) defined according to Guinebretière and coworkers (36), and one new group, called group VIII (Fig. 3). The particular phylogenetic groups were represented by 22.0%, 5.9%, 20.2%, 14.0%, 36.8%, and 1.1% of isolates under study, respectively. Group II, with the B. wiedmannii FSL W8-0169 reference strain (ST1081), contained bacilli originating mainly from Poland (42.7%) and Kazakhstan (47.6%), while other geographic locations were represented by less than 5.0% of isolates. Group III, apart from the B. cereus ATCC 10987 (ST32), B. cereus ATCC 4342 (ST38), emetic B. cereus F4810/72 (ST26), and B. anthracis ATCC 14578 (ST1) reference strains, was represented by Kenyan (40.9%) and Burkinabe (31.7%) isolates, whereas bacilli from Poland (13.6%), Kazakhstan (9.1%), and Argentina (4.5%) had smaller contributions in this group. The B. thuringiensis HD1 (ST10), B. thuringiensis HD73 (ST8), and B. cereus ATCC 14579 (ST4) reference strains clustered together within group IV with Kenyan (37.3%) and Argentinian (34.7%) strains, yet the contributions of Kazakh, Polish, and Burkinabe bacilli were 12%, 8%, and 8%, respectively. Group V, with the *B. toyonensis* BCT-7112 reference strain (ST111), contained isolates originating mostly from Poland (71.2%) and Argentina (21.2%). Group VI, with the B. weihenstephanensis DSMZ 11821 (ST447) and B. mycoides ATCC 6462 (ST116) reference strains, also comprised mainly Polish isolates (94.9%), while group VIII, with the *B. luti* TD41 (ST764) reference strain, consisted of four Kenyan isolates only. None of the environmental strains were clustered with B. pseudomycoides WSBC 10360 (ST87) and DSMZ 12442 (ST1305) within group I, as well as with *B. cytotoxicus* DSMZ 22905 (ST930) within group VII, according to the scheme elaborated by Guinebretière and coworkers (36).

The *cytK-2* B. cereus sensu lato positive isolates accounted for 90.9% and 98.7% of strains within groups III and IV, respectively, but only for 24.4% and 1.5% within groups II and VI, respectively (Fig. 3). In contrast, strains harboring the *hblA* and *nheA* genes clustered into all phylogenetic groups, with the exception of group VIII, which contained only four Kenyan *nheA*-positive isolates (Fig. 3). The percentages of *hblA*- and *nheA*-positive strains were as follows: 76.8% and 81.7% within group II, 47.8% and 100% within group III, 97.3% and 88.0% within group IV, 78.8% and 100% within group V, and 48.5% and 94.1% within group VI, respectively.

**FIG 4:**
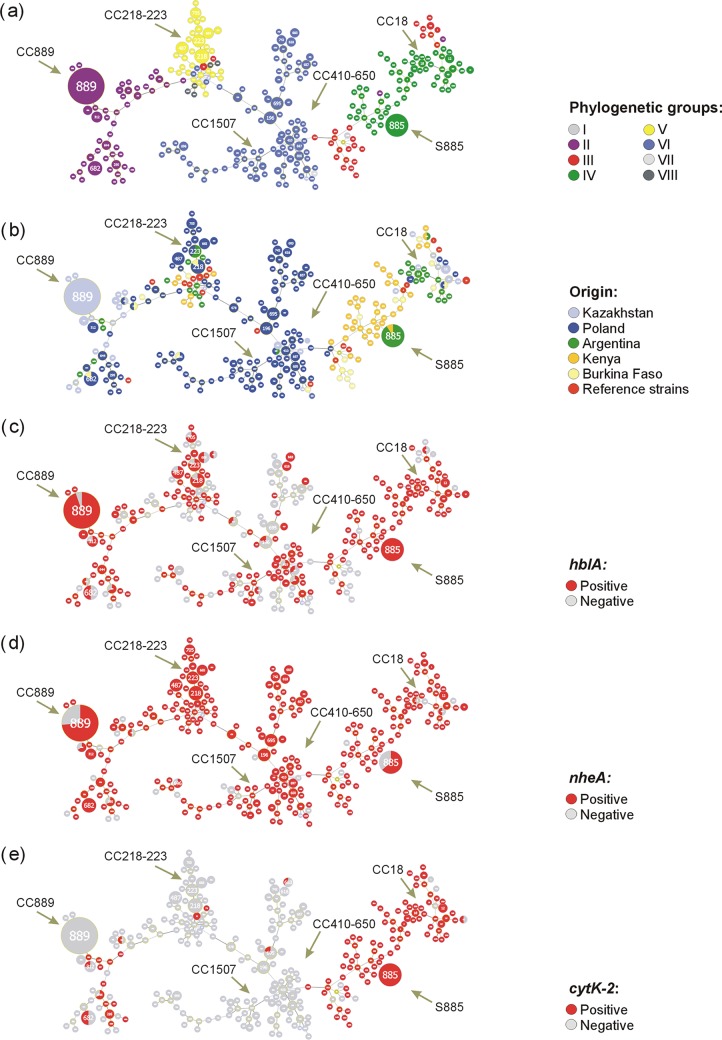
Minimum spanning tree-like structure (MST) with the largest clonal complexes (CCs) present in B. cereus sensu lato, including their phylogenetic relatedness (a), origins (b), and enterotoxic potential (c to e). The STs were assigned to CCs using PHYLOViZ v2.0 with the goeBURST algorithm and 1,000 bootstrap resampling. The relative size of the circles indicates their prevalence among the studied isolates (*n* = 396). The CCs are defined as single locus variants (SLVs) of two or more independent isolates that shared identical alleles at six or seven loci. The CCs and singletons (S) are named based on the ST assigned as a founder genotype(s) of the complex/singleton. Detailed information about participation of individual STs in clonal complexes and singletons is provided in Table S1 in the supplemental material.

The goeBURST approach enabled the assignment of isolates to 35 clonal complexes (CCs) containing 114 STs (*n* = 194) and 141 singletons (S) (*n* = 178) (Table S1). All CCs were named after putative founders of STs. Using the goeBURST Full minimum spanning tree (MST) algorithm, a minimum spanning tree-like structure was drawn, where the largest clonal complexes were indicated (Fig. 4a to e). It is not surprising that STs representing phylogenetically closely related strains form separate clusters (Fig. 4a). However, we also showed a strong link between strains originating from the same geographical location (Fig. 4b). In addition, while *hblA*- and *nheA*-positive strains were scattered throughout the MST-like tree, *cytK-2*-positive isolates appeared to cluster together (Fig. 4c to e). The CC18 complex (5 STs) consisted of six *cytK-2*-positive strains from Argentina that clustered in group IV, while other clonal complexes (e.g., CC1510, CC1512, and CC1494) had ≤5 *cytK*-2-positive strains isolated from one (e.g., CC1510 with Polish isolates) or more (e.g., CC1512 and CC1494) geographic locations. It is worth noting that one of the singletons (S885, group IV) contained 11 *cytK-2*-positive strains (ST885) originating from Argentina and Kenya (Fig. 4c). However, the most numerous complexes, CC410-650 (15 STs, *n* = 26) and CC218-223 (14 STs, *n* = 26), included *cytK*-negative strains originating mostly from Poland and clustered in groups VI and V on the phylogenetic tree, respectively. In addition, some *cytK*-negative Polish isolates from group VI clustered together into the minor complex CC1507 (7 STs, *n* = 9). CC889 (6 STs, *n* = 24) contained mostly *cytK*-negative strains from Kazakhstan that clustered in group II. However, there were also potentially cytotoxic strains (AY9-1, AR2-1, and AR2-3) found in this group.

### Enterotoxic potential of B. cereus sensu lato representing different geographic locations and varied phylogenetic groups.

**FIG 5:**
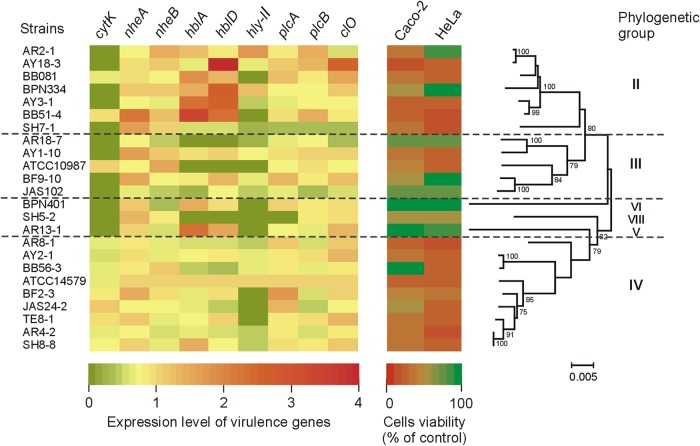
Heat map of relative expression levels of mRNAs for potentially virulent genes and *in vitro* cytotoxicity in regard to the phylogenetic relatedness of B. cereus sensu lato isolates. The phylogenetic tree for 24 strains was constructed based on MLST results as described in the legend to Fig. 3. Relative expression levels of mRNAs of particular genes were calculated using Pfaffl’s method with *udp* (uridine phosphorylase) as a reference gene and compared with calibrator B. cereus ATCC 14579. Values lower than 0.05 were considered a lack of expression. The heat map was created using conditional formatting in Excel 2010 (Microsoft). In the relative expression level bar, green indicates no expression or lack of particular genes, while dark red indicates a high expression level of mRNAs of particular genes. In the cell viability bar, green indicates high cell viability (low *in vitro* cytotoxicity of the strain), while dark red indicates low cell viability (high *in vitro* cytotoxicity of the strain). *nheA* and *nheB*, genes encoding nonhemolytic enterotoxin (NHE); *hblA* and *hblD*, genes encoding hemolysin BL (HBL); *hlyII*, gene encoding hemolysin II; *plcA*, gene encoding 1-phosphatidylinositol phosphodiesterase; *plcB*, gene encoding phospholipase C; *clO*, gene encoding thiol-activated cytolysin O.

To identify enterotoxic potential within environmental bacilli, altogether, 22 isolates representing various phylogenetic groups and originating from different geographical locations were chosen for the cytotoxicity assays. The highest *in vitro* cytotoxicity to Caco-2 and HeLa cells was demonstrated by the strains clustered within groups II and IV. The average viability of Caco-2 and HeLa cells treated with supernatants of strains clustered in group II was 31.9% and 39.9% and was 44.0% and 22.5% for that of strains within group IV, respectively. Moderate cytotoxicity was noted for strains within group III, for which the viability of Caco-2 and HeLa cells was 60.5% and 64.5%, respectively (Fig. 5 and Table S2). The Spearman correlation coefficient between Caco-2 and HeLa cell viabilities indicates positive relationship between variables (*r_s_* = 0.59, *P* = 0.002). However, the *t* test showed statistically significant differences only in HeLa cell viability between strains belonging to phylogenetic group III versus IV (*t* = 3.98, *P* = 0.001), while other observed differences were not statistically significant at a *P* value of <0.05. The viabilities of Caco-2 and HeLa cells treated with the supernatant of SH5-2 (group VIII) with neither the *hblA* nor the *cytK* and *hlyII* genes were 56.0% and 57%, respectively. In contrast, supernatants of strains belonging to groups V (AR13-1) and VI (BPN401) without the *cytK* and *hlyII* genes caused a minimal decrease in Caco-2 (8.0% and 3.0%, respectively) and HeLa (15.0% and 7.0%, respectively) viability. The results of Caco-2 and HeLa cell viability expressed as a percentage of the control are summarized in Table S2 and visualized in Fig. 5.

**FIG 6:**
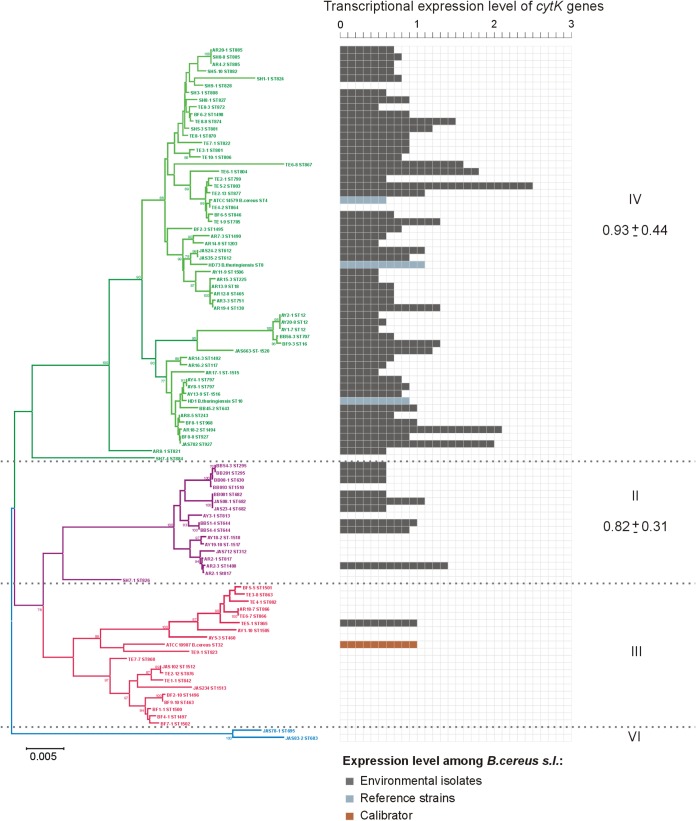
Transcriptional expression of cytotoxin K-encoding genes among the B. cereus sensu lato *cytK-2*-positive isolates. The phylogenetic tree of 94 B. cereus sensu lato strains was constructed based on seven concatenated housekeeping loci (MLST method) as described in the legend for Fig. 3. Relative mRNA expression levels of the *cytK-2* gene were calculated using Pfaffl’s method with *udp* (uridine phosphorylase) as the reference gene and compared with that in calibrator B. cereus ATCC 10987. Values lower than 0.05 were considered a lack of expression. Mean values (± standard deviation [SD]) are given on the right.

The above-described results indicate that while the most of the identified genes were expressed, the relative value of the *cytK-2* expression in some isolates from phylogenetic groups II and III was below a detectable level. Therefore, transcription of the *cytK-2* gene under standard laboratory conditions was examined for 94 bacilli from all geographic locations (Table S1). Expression values lower than 0.05 were considered a lack of expression. Altogether, 89% (17/19) of Argentinian isolates, 50% (6/12) of Burkinabe strains, 58% (7/12) of Kazakh bacilli, 63% (19/30) of Kenyan isolates, and 67% (14/21) of Polish bacilli transcribed *cytK-2* to mRNA. The mean level of *cytK-2* expression (EL*_cytK_*) in relation to B. cereus ATCC 10987 was 0.77 ± 0.47 in Argentinian, 0.94 ± 0.21 in Burkinabe, 0.66 ± 0.17 in Kazakh, 1.08 ± 0.5 in Kenyan, and 0.92 ± 0.39 in Polish isolates. We showed that *cytK-2* was expressed mostly in strains representing group IV (95% strains; EL*_cytK_* = 0.93 ± 0.44) and group II (56% strains; EL*_cytK_* = 0.82 ± 0.31). In group III, only one Kenyan strain, TE8-1, transcribed this gene, with an EL*_cytK_* =1.01, while no expression was detected in isolates of group VI (Fig. 6). A *t* test for two independent means showed that differences between EL*_cytK_* values within groups II and III were not significant at a *P* value of <0.05. However, the χ^2^ test indicated significant differences in proportions of *cytK-2*-expressing bacilli in particular phylogenetic groups (II to IV) at a *P* value of <0.05.

Since the expression of most virulence genes in B. cereus sensu lato is activated by a pleiotropic regulator system, PlcR-PapR (31, 40), we analyzed the genes encoding the regulator among 22 *de novo* sequenced isolates and two references. The PlcR- and PapR-encoding genes were found among all sequenced strains. A genetic tree constructed based on the *plcR* sequences revealed the presence of three major groups among the strains with the ability to express mRNA of *cytK-2*. These *plcR* clusters correlated well with the last five carboxy-terminal amino acid motifs of PapR sequences (LPFEY, LPFEF, and MPFEF) (see Fig. S2). In addition, we identified a TATGNAN_4_TNCATA box for PlcR located 79 bp to 264 bp upstream from the *cytK-2* gene among 21 analyzed strains (data not shown; for accession numbers, see Table S1). Instead, in BB51-4, a box located 88 bp upstream from the target sequence with a replacement of A_2_ by G was observed; however, the replacement had no effect on *cytK-2* transcription in this strain.

The *de novo* obtained sequences were also analyzed with regard to other virulence determinant. All B. cereus sensu lato strains possessed the *nhe* genes (*nheA*, *nheB*, and *nheC*) as well as *hlyIII*, *plcB*, *smase*, and *clO* genes, encoding nonhemolytic enterotoxin (NHE), hemolysin III, phospholipase C, sphingomyelinase C, and thiol-activated cytolysin, respectively. All three genes (*hblA*, *hblC*, and *hblD*) of the *hbl* operon encoding the tripartite hemolysin BL (HBL) were present in 91%, while the *hlyII* gene for hemolysin II was noted among 64% of the environmental strains under study (Table S2). The transcription of particular genes (except *nheC*, *hblC*, *hlyIII*, and *smase*, for technical reasons) among *de novo* sequenced bacilli was confirmed (Fig. 6 and Table S2). Lack of the gene was considered a lack of expression. The average expression levels (EL*_av_*) of most genes within phylogenetic groups II, III, and IV were of similar degrees. However, the EL*_av_* of the *cytK-2* gene was at a very low level in group II (0.23 ± 0.15) and group III (0.01 ± 0.00), whereas it increased within group IV to 0.76 ± 0.06. The EL*_av_* of the *hblA* gene within group II (1.63 ± 0.37) decreased in group III and group IV to 0.51 ± 0.22 and 0.74 ± 0.10, respectively. Similarly, the EL*_av_* of the *hblD* gene in group II was 2.06 ± 0.48, while in group III and IV, it was 0.77 ± 0.31 and 0.70 ± 0.09, respectively. The Spearman correlation coefficient analysis between relative mRNA expression levels and Caco-2 or HeLa cell viability were conducted. A statistically significant negative correlation (*r_s_* = −0.47, *P* = 0.02) was shown only between *cytK-2* mRNA expression level and HeLa cell viability.

## DISCUSSION

B. cereus sensu lato spores are widely distributed around the world and have been found in soil, with concentrations ranging from 2 × 10^4^ to 5 × 10^5^ CFU g^−1^ (22, 34, 41–43). In consequence, diverse raw food ingredients, such as vegetables, grains, herbs, and spices, are often contaminated with these bacilli (3). In this study, we showed differences in the geographical distributions of potential enterotoxigenic B. cereus sensu lato strains isolated from soil originating from Poland (Bialowieza National Park, Biebrza National Park, and a farm in Jasienowka), Kazakhstan (a park in Almaty), Argentina (a park in Buenos Aires), Africa, e.g., Burkina Faso (a public place near urban Bangr Weogo Park in Ouagadougou), and Kenya (car parks in Tsavo East National Park and Shimba Hills National Reserve) (Fig. 1). Strains harboring *cytK-2*, *nheA*, and *hblA* simultaneously dominated in geographic regions with an arid hot climate (Africa), while only a few isolates were noted in Poland, which has a continental cold climate (Fig. 2 and 4). Africa and Latin America are known as areas with a high risk of traveler’s diarrhea. Available data suggest Escherichia coli as a major causative agent of this disorder, followed much less frequently by *Campylobacter* spp., *Salmonella*, *Shigella* spp., *Aeromonas* spp., and *Vibrio* spp (44). However, in most countries, diarrhea caused by B. cereus is not a notifiable disease; thus, case reports are extremely limited.

Although enterotoxigenic and enterotoxic potentials are highly variable between strains of B. cereus sensu lato and differ significantly due to their nature associated with the harbored genes but also the condition of the host and the food content (19), our phylogenetic study showed the existence of potential B. cereus pathotypes connected with some phylogenetic groups, especially group IV (Fig. 3 and 6). These isolates are characterized by (i) the presence of a CytK-2-encoding gene and other genetic determinants potentially associated with virulence, (ii) a high level of *cytK-2* gene expression in these isolates, and (iii) their high cytotoxic activity assessed in the *in vitro* cytotoxicity assays with the use of the HeLa and Caco-2 cells. Indeed, in group IV, 83.7% of isolates harbored all three genes tested, *cytK*, *nheA*, and *hblA*. It was shown that this phylogenetic group contains mesophilic (growth range 10 to 45°C) bacteria (36, 37). Nevertheless, it is worth noting that also within group III, we found a high percentage of strains harboring *cytK-2*, but the gene was not transcribed (see below). Interestingly, these groups were dominated either (i) by strains isolated from Africa, with an arid hot steppe and a wet savanna (group III) or (ii) by bacilli from Argentina, with a temperate climate with hot summer, and Africa (group IV) (Fig. 3). The common occurrence of the enterotoxin-encoding genes among strains isolated from “hot” regions (Fig. 4) and clustered in mesophilic phylogenetic groups (Fig. 3) seems to result from environmental adaptation. Such an ecologically similar group of bacteria closely related at the molecular level evolve and proliferate as a result of acquired genetic determinants or favorable mutations that confer selective advantages in a particular niche or ecosystem (22, 45). From this perspective, we suggest that *cytK-2* variants could be acquired via horizontal gene transfer during the evolution of B. cereus sensu lato. Indeed, our results are in accordance with the suspected first appearance of *cytK-2* published by Böhm and coworkers (32). It seems that a common ancestor of groups II to IV might have acquired this toxin gene via lateral gene transfer from the ancestor of the *B. cytotoxicus* lineage prior to splitting into clusters II, III, and IV. Then, the *cytK-2* gene may have been lost in some strains in psychrotolerant group II (7 to 40°C), which contains bacilli mostly from geographic locations with a cold continental climate (Poland and Kazakhstan), or occasionally acquired by a recent horizontal gene transfer by Polish isolates clustered in psychrotolerant group VI (5 to 37°C) (Fig. 3).

Expression of most virulence genes, including *cytK*, is activated by a pleiotropic regulator, PlcR, which depends on the presence of a quorum-sensing effector, PapR. This signaling peptide is exported by the bacterial cell, processed, and reimported into the cell, where it interacts with PlcR, facilitating its binding to a consensus sequence of the target genes defined as TATGNAN_4_TNCATA (30, 31, 40, 46). Then, toxins are secreted using the general secretory (Sec) pathway via membranes to act at the target host cell (47). We showed that *cytK-2* is expressed among strains belonging to groups II and IV, while no expression was found among strains belonging to group III (except strain TE8-1 from Tsavo East National Park in Kenya). In the isolates classified to clades II and IV, PapR pentapeptides with specific final five carboxy-terminal amino acid motifs (LPFEY, LPFEF, and MPFEF) seem to be responsible for activation of the quorum-sensor PlcR (see Fig. S2 in the supplemental material). Indeed, Slamti and Lereclus (40) indicated that specific PlcR-PapR pairs (with LPFEY or LPFEF motifs) are active quorum-sensing systems of the B. cereus group.

It was shown that the CytK-2 variant is less toxic to human intestinal Caco-2 cells and Vero cells than CytK-1 (18). Yet, the contribution of CytK-2 to diarrheal syndrome has been questioned in a recent study (48); in another investigation, it was demonstrated that this enterotoxin may contribute to intestinal infections, but the CytK-2 toxicity of some B. cereus strains is lower than that of CytK-1 (29). Our results indicate that strains clustered in group IV, which expressed most enterotoxin determinants, including *cytK-2*, were more cytotoxic to Caco-2 and HeLa cells than strains clustered in groups III, V, and VI, with no expression of *cytK-2* or without the CytK-2-encoding gene (Fig. 5; Table S2). Lack of a statistically significant relationship between gene expression and cell viability suggests that many genetic determinants can play important roles in B. cereus diarrhea. Naturally, the diarrheal syndrome can be caused simultaneously by a number of different toxins, such as nonhemolytic enterotoxin NHE, hemolysin II, hemolysin III, phospholipases, and various proteases (3, 49).

Certainly, B. cereus sensu lato strains pose a serious risk for human health; hence, rapid testing methods for the identification of enterotoxins in food matrices are greatly needed. At present, only a few commercial enzyme immunoassays are available, for example, the BCET-RPLA kit (Oxoid, Ltd.), TECRA BDE-VIA (Bioenterprises Pty, Australia), and Duopath test (Merck KGaA, Darmstadt, Germany) (21, 33); however, none of them detect whether a functional cytotoxin K is present. We showed that strains clustered in groups II and IV are more cytotoxic to epithelial cells than other bacteria in the B. cereus group. The *cytK-2* contribution to cytotoxicity cannot be excluded, especially in group IV. Thus, the detection of the CytK enterotoxin should be a supporting strategy for the identification of diarrheal risk caused by B. cereus sensu lato together with cultivation of these isolates under conditions simulating intestinal host condition, e.g., in the presence of Caco-2 cells (19).

In conclusion, B. cereus sensu lato strains from individual geographic locations grouped together into different thermal phylogenetic groups. Strains with high enterotoxigenic potential dominated in geographic regions with an arid hot climate (Africa), while only a few *cytK-2*-positive isolates were noted in Poland, which has a continental cold climate. In addition, B. cereus sensu lato harboring a CytK-2-encoding gene clustered together mainly within mesophilic groups III and IV, which might be due to the adaptation to “hot” geographic regions. Cytotoxicity to epithelial cells associated with an increased expression level of (i) the *hbl* operon or (ii) the *cytK-2* gene was observed mainly in group II or IV, respectively.

## MATERIALS AND METHODS

### B. cereus sensu lato isolates and reference strains.

Five populations of B. cereus sensu lato, originating from (i) a town park in Buenos Aires, Argentina (*n* = 201), (ii) a town park in Almaty, Kazakhstan (*n* = 200), (iii) car parks within Tsavo East National Park and Shimba Hills National Reserve in Kenya (*n* = 211), (iv) a public place near urban Bangr Weogo Park in Ouagadougou in Burkina Faso (*n* = 100), and (v) two national parks (Bialowieza National Park and Biebrza National Park) and a farm in Jasienowka in northeastern Poland (*n* = 300), were investigated in this study. B. cereus sensu lato isolation and identification were performed according to the methods of Drewnowska and Swiecicka (22). To differentiate B. thuringiensis strains from other species of B. cereus sensu lato, parasporal crystals were investigated using phase-contrast microscopy (Olympus BX61; Olympus, Tokyo, Japan). Isolates growing at 7°C and containing the *cspA* psychrotolerance motif (^4^ACAGTT^9^) (50) were reclassified to B. weihenstephanensis (to avoid confusion, we keep the original classification; however, according to the latest classification, all B. weihenstephanensis strains should be considered B. mycoides [11]). Because most of the routine methods described so far are unable to discriminate between B. cereus sensu lato species, such as B. cereus, *B. toyonensis* or B. wiedmannii, in an accurate and reliable way, we decided to mark them as B. cereus sensu lato. The Argentinian, Kazakh, and Kenyan part of the collection was isolated during a previous study by Kaminska and coworkers (41), whereas the Polish isolates were isolated by Drewnowska and Swiecicka (22).

B. cereus ATCC 10987, B. cereus ATCC 14579 (American Type Culture Collection), B. thuringiensis HD73, B. thuringiensis HD1 (Bacillus Genetic Stock Center [BGSC]), B. weihenstephanensis DSMZ 11821 (German Collection of Microorganisms and Cell Cultures), *B. cytotoxicus* NVH883-00, and *B. cytotoxicus* NVH391-98 were used as reference strains. In addition, genome sequences of 24 type strains as references were obtained from the NCBI database (https://www.ncbi.nlm.nih.gov/). Detailed information about all strains used in this study is available in Table S1 in the supplemental material.

### Detection of virulence genes.

Genomic DNA was extracted from an overnight culture of an isolate in Luria-Bertani (LB) broth using a DNeasy blood and tissue kit (Qiagen GmbH, Hilden, Germany) in accordance with the manufacturer’s protocol for Gram-positive bacteria. To amplify the *nheA* and *hblA* genes as well as *cytK-1* and *cytK-2* forms, the primer pairs listed in Table S3 were used as recommended in other works (50–52). Due to the lack of full specificity of CKF/CKR for many *cytK-2* target genes, the *cytK* sequences from the NCBI data bank (https://www.ncbi.nlm.nih.gov/) were used to design an alternative pair of primers (CytK2F/CytK2R). All B. cereus sensu lato isolates (*n* = 1,012) were screened for the presence of three virulence genes. The final PCR mixture (15 μl) contained 7.5 μl of StartWarm HS-PCR mix (A&A Biotechnology, Gdynia, Poland), 100 ng of DNA, and 0.25 μM each primer from a particular pair. *B. cytotoxicus* NVH 391/98 and B. cereus ATCC 14579 were applied as reference strains. PCR products were separated on a 1% agarose gel with Midori Green Advance DNA stain (Nippon Genetics Europe GmbH, Düren, Germany) and a GeneRuler 1 kb DNA ladder (Thermo Fisher Scientific) and visualized using a molecular gel imager, ChemiDoc XRS+ (Bio-Rad Laboratories Inc., Hercules, CA, USA).

### Phylogenetic study and population structure analysis.

Altogether, 73 randomly chosen isolates were characterized by the MLST scheme according to the B. cereus PubMLST database (https://pubmlst.org/bcereus/) and previous work (22). Briefly, 534- to 599-bp fragments of seven housekeeping loci (*glpF*, *gmk*, *ilvD*, *pta*, *pur*, *pycA*, and *tpi)* were sequenced using an ABI3500 automated sequencer (Applied Biosystems) according to a protocol previously described (22) (Table S2). The combination of allele numbers for all seven loci for a given isolate allowed assessment of the specific sequence type (ST). New allele sequences and STs were submitted to the B. cereus PubMLST database.

For phylogenetic analysis, altogether, 372 environmental isolates and 24 references were included. A phylogenetic tree based on the concatenated loci (2,829 nucleotides) was constructed for all isolates using the neighbor-joining (NJ) method together with MEGA7 software (53). Branch quality was evaluated using 1,000 bootstrap replicates. Detailed information about the strains used in this study is given in Table S1. The STs were assigned to clonal complexes using PHYLOViZ v2.0 (54) with the goeBURST algorithm and 1,000 bootstrap resampling according to Feil and coworkers (55). Clonal complexes (CCs) were defined as single locus variants (SLVs) of two or more independent isolates that shared identical alleles at six or seven loci.

### Quantitative real-time reverse transcription-PCR of virulence genes.

RNA was isolated from strains cultured in LB broth (10 h, 30°C, 180 rpm) using a Total RNA Mini Plus kit (A&A Biotechnology) according to the manufacturer’s protocol, with some modifications as described by Drewnowska and coworkers (56). The *cytK* primers (RT-cytKF2/RT-cytKR2) (Table S3) were designed based on the conserved regions of *cytK-2* fragments sequenced during this study. Briefly, amplified products from all *cytK-2*-positive isolates (*n* = 94) were purified enzymatically using the EPPiC Fast reagent (A&A Biotechnology) and sequenced with an ABI3500 automated sequencer (Applied Biosystems) using a BigDye Terminator v3.1 cycle sequencing kit (Applied Biosystems) according to the manufacturer’s protocol. The *cytK* sequences were assembled with BioEdit Sequence Alignment Editor v7.0.5 software and identified using the BLAST algorithm (57) as well as deposited in the GenBank database (see also Table S1 for accession numbers). Primers for other virulence genes were designed based on their conserved regions sequenced during this study (for details, see “Whole-genome sequencing for virulence factor screening”) (Table S3).

The cDNA was analyzed using a StepOne Plus real-time PCR system (Applied Biosystems). Reactions were performed using a real-time PCR (RT-PCR) master mix SYBR A kit (A&A Biotechnology) in 20 μl of a reaction mixture containing 10 μl of RT-PCR master mix, 0.3 mM each forward and reverse primer, and 1.5 μl of diluted cDNA (1:10). The threshold cycle (*C_T_*) was normalized to the *C_T_* of the *udp* (uridine phosphorylase) reference gene (58) amplified from the corresponding isolate. Gene expression was calculated using Pfaffl’s method (59) in comparison with referential B. cereus ATCC 10987 and B. cereus ATCC 14579. Values lower than 0.05 were considered a lack of expression.

### *In vitro* cytotoxicity assays.

We examined the cytotoxicity of 22 environmental isolates and two reference strains on the human cervical cancer cell line HeLa (CCL-2; ATCC, Manassas, VA, USA) and the colon adenocarcinoma cell line Caco-2 (HTB-37; ATCC, Manassas, VA, USA). HeLa and Caco-2 cells were cultured under a 5% CO_2_ and water-saturated atmosphere in Dulbecco’s modified Eagle’s medium (DMEM) supplemented with 4.5 g/liter of glucose and 10% fetal bovine serum (FBS; Sigma-Aldrich), 50 U/ml penicillin, and 50 mg/ml streptomycin at 37°C. Cells were seeded at a density of 1 × 10^5^ cells/well in 24-well plates. HeLa cells were maintained to 95% to 100% confluence. Caco-2 cells were seeded on plates precoated with type I collagen and cultured for 21 days after reaching confluence. The conditions of bacterial cultures were identical to those for RNA isolation. After a 10-h incubation (30°C, 180 rpm), bacterial cultures (optical density at 600 nm [OD_600_] of ∼1.5) were centrifuged (4°C, 10 min, 7,500 × *g*), and supernatants were filtered by polyvinylidene difluoride (PVDF) syringe filters with a 0.22-μm pore size (Carl Roth GmbH & Co. KG., Karlsruhe, Germany). HeLa and Caco-2 cells were washed with Dulbecco’s phosphate-buffered saline (PBS; Sigma-Aldrich). Subsequently, cells were incubated for 1 h with bacterial supernatants, diluted 30 (HeLa cells) or 10 times (Caco-2 cells) in Hanks’ balanced salt solution (HBSS; Sigma-Aldrich), and then rinsed two times with PBS before performing assays. For each experiment, cells incubated with LB medium and diluted 30 times in HBSS served as controls. The toxicity of bacterial supernatants was determined by colorimetric detection using the 3-(4,5-dimethyl-2-thiazolyl)-2,5-diphenyl-2H-tetrazolium bromide (MTT) test (60). The cells were incubated for 1 h in 0.5 ml of PBS with 50 μl of MTT (5 mg/ml). Then, the medium was removed from the wells, and formazan crystals were dissolved in 0.5 ml of dimethyl sulfoxide (DMSO) with 0.01 ml of Sorensen’s buffer (0.1 M glycine with 0.1 M NaCl, pH 10.5). Absorbance was recorded with a Lambda E plate reader (MWG Biotech AG, Ebersberg, Germany) at a wavelength of 570 nm. Values were expressed as a percentage of those for control cells. Correlations between cell viability rates and virulence gene (mRNA) expression levels were analyzed using the Spearman’s rank correlation coefficient (SCC), which indicates positive relationship where *r_s_* is >0, while an *r_s_* value of <0 indicates negative relationship, and an *r_s_* value of 0 indicates no relationship between the ranks.

### Whole-genome sequencing for virulence factor screening.

Genomic DNA from the 22 environmental strains tested on HeLa cells was isolated as described above. The quantity was measured using both a Qubit 2.0 fluorometer with a Qubit dsDNA HS assay kit (Invitrogen, Thermo Fisher Scientific) and a 2200 TapeStation instrument with a genomic DNA ScreenTape assay (Agilent Technologies Inc., Santa Clara, CA, USA). Genomic libraries were prepared using a Nextera XT kit (Illumina Inc., San Diego, CA, USA) according to the manufacturer’s protocol and quantified by capillary electrophoresis, applying the Agilent High Sensitivity D5000 ScreenTape system (Agilent Technologies Inc.). Libraries were sequenced on a MiSeq machine (Illumina) using v2 reagents with 2 × 250-bp paired-end reads. Consequently, 81.6% to 98.8% of bases in sequencing reads had quality scores of 30 (Q30) or higher. *De novo* genome assembly was performed using CLC Genomic Workbench v5 (Qiagen GmbH). Rapid Annotation using Subsystem Technology (RAST server; https://rast.nmpdr.org/) was used for functional annotation of proteins (61).

### Data availability.

The data sets supporting this study have been deposited in the National Center for Biotechnology Information (NCBI) databases. The c*ytK-2* sequences were deposited in the GenBank nucleotide archive under accession numbers MH618405 to MH618487 (for details, see Table S1). NGS data were deposited in the NCBI database under accession numbers QKQR00000000, QOZW00000000, QORY00000000, QORZ00000000, QNUD00000000, QOSC00000000, QOZX00000000, QPAL00000000, QNUC00000000, QPEM00000000, QPEK00000000, QNUB00000000, QOSA00000000, QPEL00000000, QPET00000000, QOSB00000000, QNUA00000000, QOSD00000000, CP031062 to CP031064, CP031065 to CP031067, CP031068 to CP031070, and CP031071 to CP031077 (for details see Table S2).

## Supplementary Material

Supplemental file 1
