# Supplementary material for: Potential Enterotoxicity of Phylogenetically Diverse Bacillus cereus Sensu Lato Soil Isolates from Different Geographical Locations
Source: Appl Environ Microbiol. 2020 May 19;86(11):e03032-19. doi: 10.1128/AEM.03032-19 (PMC7237779; doi:10.1128/AEM.03032-19)
Supplement: Supplemental file 1 [file AEM.03032-19-s0001.pdf]

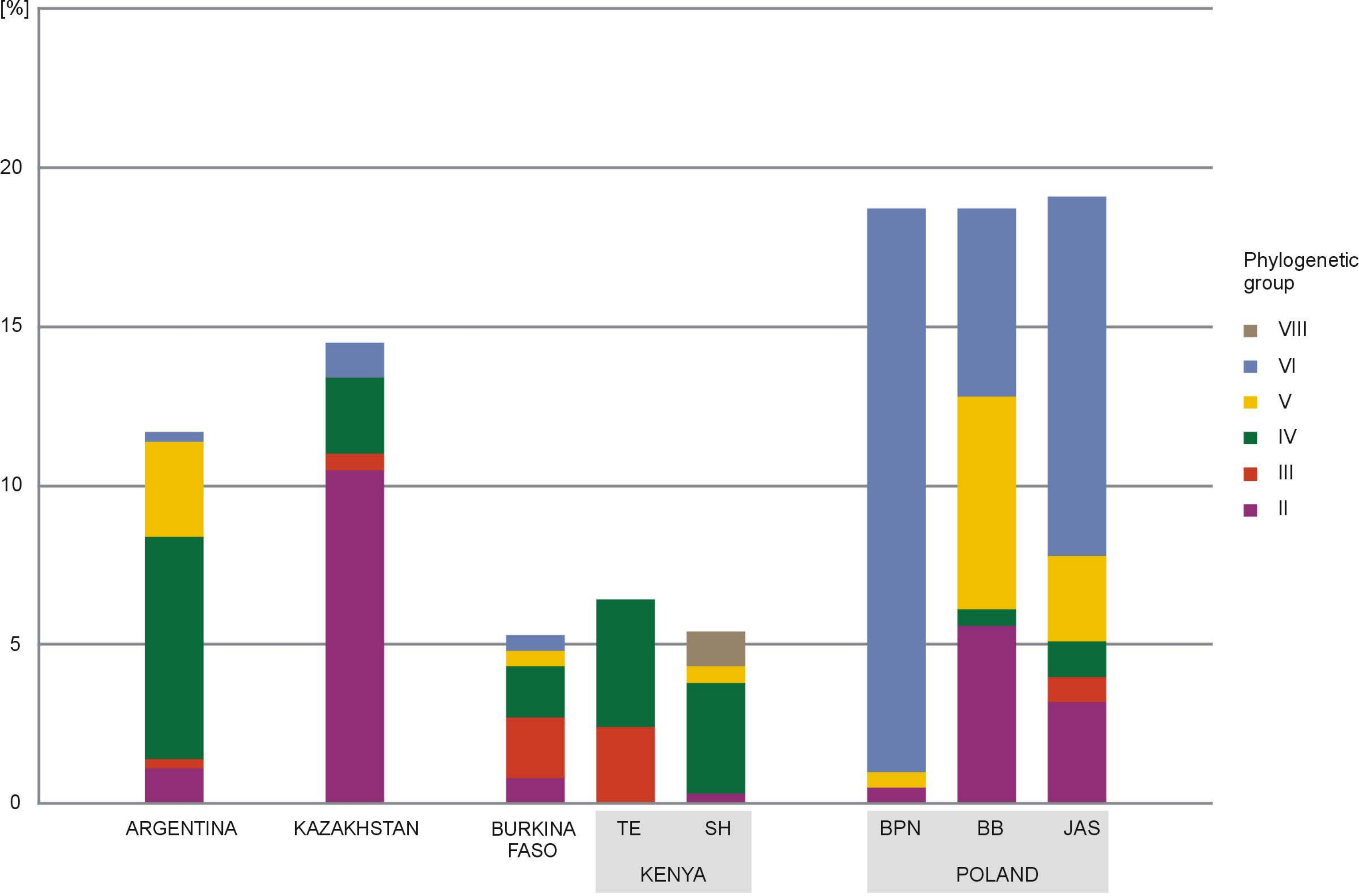

Fig.S1. Distribution of isolates from particular origins within phylogenetic groups

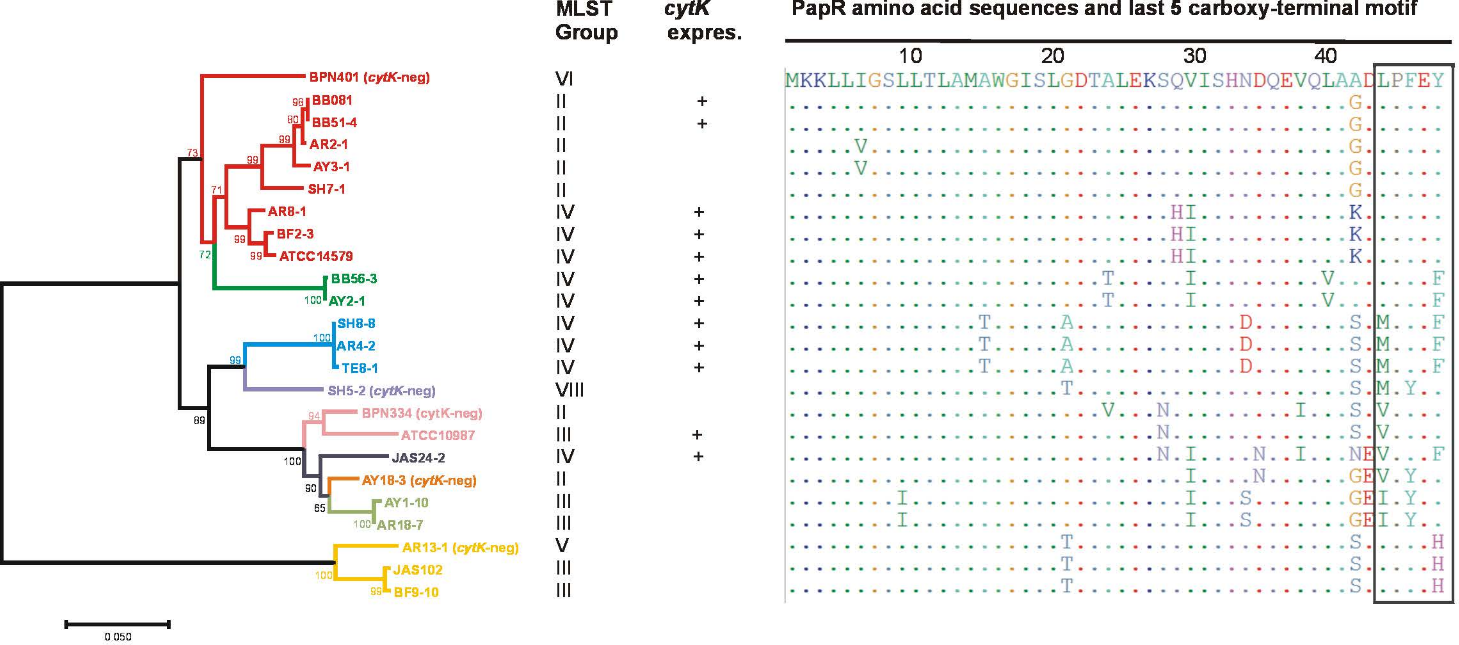

Fig.S2. The *plcR* tree obtained with Neighbor Joining (NJ) method on 24 *B.cereus s.l.* strains compared with PapR amino acid sequences.

Table S1. Characteristic of *B. cereus s.l.* isolates originating from different geographic locations and references used in this study.

Strain	Species[a]	Origin	PCR				*cytK-2*	$EL_{cytK\text{-}2}$ [b]	ST[c]	MLST	CC[d]	Reference for MLST data
			hblA	*nheA*	*cytK-1*	*cytK-2*	GenBank Acces. No.			Group		
AR 1-1	*B. cereus* s.l.	ARGENTINA	-	+	-	-			480	V	S	Kaminska *et al.* (2015)
AR 1-2	*B. cereus* s.l.	ARGENTINA	-	+	-	-						
AR 1-3	*B. cereus* s.l.	ARGENTINA	-	-	-	-						
AR 1-4	*B. cereus* s.l.	ARGENTINA	+	-	-	-						
AR 1-5	*B. cereus* s.l.	ARGENTINA	+	+	-	+						
AR 1-6	*B. cereus* s.l.	ARGENTINA	+	-	-	+	MH618405	0.72	18	IV	CC18	this study
AR 1-7	*B. cereus* s.l.	ARGENTINA	+	+	-	-						
AR 1-8	*B. cereus* s.l.	ARGENTINA	-	+	-	-						
AR 1-9	*B. cereus* s.l.	ARGENTINA	-	+	-	-						
AR 1-10	*B. cereus* s.l.	ARGENTINA	+	+	-	-			484	V	S	Kaminska *et al.* (2015)
AR 2-1	*B. cereus* s.l.	ARGENTINA	+	+	-	+	MH618406	0.00	817	II	CC889	Kaminska *et al.* (2015)
AR 2-2	*B. cereus* s.l.	ARGENTINA	+	-	-	-						
AR 2-3	*B. cereus* s.l.	ARGENTINA	+	-	-	+	MH618407	1.43	1488	II	CC889	this study
AR 2-4	*B. cereus* s.l.	ARGENTINA	+	+	-	-						
AR 2-5	*B. cereus* s.l.	ARGENTINA	+	-	-	-						
AR 2-6	*B. cereus* s.l.	ARGENTINA	+	+	-	+						
AR 2-7	*B. cereus* s.l.	ARGENTINA	+	+	-	+						
AR 2-8	*B. cereus* s.l.	ARGENTINA	+	+	-	-						
AR 2-9	*B. cereus* s.l.	ARGENTINA	+	-	-	-						
AR 2-10	*B. cereus* s.l.	ARGENTINA	+	+	-	-						
AR 3-1	*B. cereus* s.l.	ARGENTINA	+	+	-	-			818	V	S	Kaminska *et al.* (2015)
AR 3-2	*B. cereus* s.l.	ARGENTINA	+	-	-	-						
AR 3-3	*B. cereus* s.l.	ARGENTINA	+	+	-	+	MH618408	0.67	751	IV	CC18	this study
AR 3-4	*B. cereus* s.l.	ARGENTINA	+	+	-	-						
AR 3-5	*B. cereus* s.l.	ARGENTINA	+	+	-	-						
AR 3-6	*B. cereus* s.l.	ARGENTINA	+	+	-	-						
AR 3-7	*B. cereus* s.l.	ARGENTINA	+	+	-	-						
AR 3-8	*B. cereus* s.l.	ARGENTINA	+	+	-	+						
AR 3-9	*B. cereus* s.l.	ARGENTINA	+	+	-	+						
AR 3-10	*B. cereus* s.l.	ARGENTINA	+	+	-	-						
AR 4-1	*B. cereus* s.l.	ARGENTINA	+	+	-	+			885	IV	S	Kaminska *et al.* (2015)
AR 4-2	*B. cereus* s.l.	ARGENTINA	+	+	-	+	MH618409	0.66	885	IV	S	Kaminska *et al.* (2015)
AR 4-3	*B. cereus* s.l.	ARGENTINA	+	-	-	+			885	IV	S	Kaminska *et al.* (2015)
AR 4-4	*B. cereus* s.l.	ARGENTINA	+	+	-	+			885	IV	S	Kaminska *et al.* (2015)
AR 4-5	*B. cereus* s.l.	ARGENTINA	+	-	-	+			885	IV	S	Kaminska *et al.* (2015)
AR 4-6	*B. cereus* s.l.	ARGENTINA	+	+	-	+			885	IV	S	Kaminska *et al.* (2015)
AR 4-7	*B. cereus* s.l.	ARGENTINA	+	-	-	+			885	IV	S	Kaminska *et al.* (2015)
AR 4-8	*B. cereus* s.l.	ARGENTINA	+	+	-	+			885	IV	S	Kaminska *et al.* (2015)
AR 4-9	*B. cereus* s.l.	ARGENTINA	+	+	-	-						
AR 4-10	*B. cereus* s.l.	ARGENTINA	+	-	-	+			885	IV	S	Kaminska *et al.* (2015)
AR 5-1	*B. cereus* s.l.	ARGENTINA	+	+	-	-			819	V	CC218-223	Kaminska *et al.* (2015)
AR 5-2	*B. cereus* s.l.	ARGENTINA	+	+	-	-						
AR 5-3	*B. cereus* s.l.	ARGENTINA	+	-	-	+						
AR 5-4	*B. cereus* s.l.	ARGENTINA	+	+	-	+						
AR 5-5	*B. cereus* s.l.	ARGENTINA	+	+	-	-						
AR 5-6	*B. cereus* s.l.	ARGENTINA	+	-	-	-						
AR 5-7	*B. cereus* s.l.	ARGENTINA	+	+	-	-						
AR 5-8	*B. cereus* s.l.	ARGENTINA	+	+	-	-						
AR 5-9	*B. cereus* s.l.	ARGENTINA	+	+	-	+			210	IV	S	this study
AR 5-10	*B. cereus* s.l.	ARGENTINA	+	+	-	-						
AR 6-1	*B. cereus* s.l.	ARGENTINA	+	+	-	-			223	V	CC218-223	Kaminska *et al.* (2015)
AR 6-2	*B. cereus* s.l.	ARGENTINA	+	+	-	-						
AR 6-3	*B. cereus* s.l.	ARGENTINA	+	+	-	+			1489	IV	S	this study
AR 6-4	*B. cereus* s.l.	ARGENTINA	+	+	-	-						
AR 6-5	*B. cereus* s.l.	ARGENTINA	+	+	-	-						
AR 6-6	*B. cereus* s.l.	ARGENTINA	+	+	-	-						
AR 6-7	*B. cereus* s.l.	ARGENTINA	+	+	-	-						
AR 6-8	*B. cereus* s.l.	ARGENTINA	+	+	-	-						
AR 6-9	*B. cereus* s.l.	ARGENTINA	+	+	-	+						
AR 6-10	*B. cereus* s.l.	ARGENTINA	+	-	-	-						
AR 7-1	*B. cereus* s.l.	ARGENTINA	+	+	-	-			820	V	CC218-223	Kaminska *et al.* (2015)
AR 7-2	*B. cereus* s.l.	ARGENTINA	+	+	-	-						
AR 7-3	*B. cereus* s.l.	ARGENTINA	+	+	-	+	MH618410	0.60	1490	IV	S	this study
AR 7-4	*B. cereus* s.l.	ARGENTINA	-	+	-	-						
AR 7-5	*B. cereus* s.l.	ARGENTINA	+	+	-	+						
AR 7-6	*B. cereus* s.l.	ARGENTINA	+	+	-	-						
AR 7-7	*B. cereus* s.l.	ARGENTINA	-	+	-	-						
AR 7-8	*B. cereus* s.l.	ARGENTINA	+	+	-	-						
AR 7-9	*B. cereus* s.l.	ARGENTINA	+	+	-	-						
AR 7-10	*B. cereus* s.l.	ARGENTINA	+	+	-	-						
AR 8-1	*B. cereus* s.l.	ARGENTINA	+	+	-	+	MH618411	0.61	821	IV	S	Kaminska *et al.* (2015)
AR 8-2	*B. cereus* s.l.	ARGENTINA	+	-	-	+						
AR 8-3	*B. cereus* s.l.	ARGENTINA	+	-	-	-						
AR 8-4	*B. cereus* s.l.	ARGENTINA	+	+	-	-						
AR 8-5	*B. cereus* s.l.	ARGENTINA	+	-	-	+	MH618412	0.67	243	IV	CC1494	this study
AR 8-6	*B. cereus* s.l.	ARGENTINA	+	-	-	-						
AR 8-7	*B. cereus* s.l.	ARGENTINA	+	+	-	-						
AR 8-8	*B. cereus* s.l.	ARGENTINA	+	+	-	-						
AR 8-9	*B. cereus* s.l.	ARGENTINA	+	+	-	-						
AR 8-10	*B. cereus* s.l.	ARGENTINA	+	-	-	-						
AR 9-1	*B. cereus* s.l.	ARGENTINA	+	+	-	-			223	V	CC218-223	Kaminska *et al.* (2015)
AR 9-2	*B. cereus* s.l.	ARGENTINA	+	+	-	-						
AR 9-3	*B. cereus* s.l.	ARGENTINA	+	+	-	-			223	V	CC218-223	Kaminska *et al.* (2015)
AR 9-4	*B. cereus* s.l.	ARGENTINA	+	+	-	-						
AR 9-5	*B. cereus* s.l.	ARGENTINA	+	+	-	-						
AR 9-6	*B. cereus* s.l.	ARGENTINA	+	+	-	-						
AR 9-7	*B. cereus* s.l.	ARGENTINA	+	+	-	-						
AR 9-8	*B. cereus* s.l.	ARGENTINA	+	+	-	-						
AR 9-9	*B. cereus* s.l.	ARGENTINA	+	+	-	-						
AR 9-10	*B. cereus* s.l.	ARGENTINA	+	+	-	-						
AR 10-1	*B. cereus* s.l.	ARGENTINA	+	+	-	-			1491	V	CC218-223	this study
AR 10-2	*B. cereus* s.l.	ARGENTINA	+	+	-	-						
AR 10-3	*B. cereus* s.l.	ARGENTINA	+	+	-	-						
AR 10-4	*B. cereus* s.l.	ARGENTINA	+	+	-	-						
AR 10-5	*B. cereus* s.l.	ARGENTINA	+	+	-	-						
AR 10-6	*B. cereus* s.l.	ARGENTINA	+	+	-	-						
AR 10-7	*B. cereus* s.l.	ARGENTINA	+	+	-	-						
AR 10-8	*B. cereus* s.l.	ARGENTINA	+	+	-	-						
AR 10-9	*B. cereus* s.l.	ARGENTINA	+	+	-	-						
AR 10-10	*B. cereus* s.l.	ARGENTINA	+	+	-	-						

AR 11-1	*B. cereus s.l.*	ARGENTINA	+	+	-	-						
AR 11-2	*B. cereus s.l.*	ARGENTINA	+	+	-	-						
AR 11-3	*B. cereus s.l.*	ARGENTINA	+	+	-	-						
AR 11-4	*B. cereus s.l.*	ARGENTINA	+	+	-	-						
AR 11-5	*B. cereus s.l.*	ARGENTINA	-	+	-	-						
AR 11-6	*B. cereus s.l.*	ARGENTINA	+	+	-	-						
AR 11-7	*B. cereus s.l.*	ARGENTINA	+	+	-	-						
AR 11-8	*B. cereus s.l.*	ARGENTINA	+	+	-	-			1499	V	S	this study
AR 11-9	*B. cereus s.l.*	ARGENTINA	+	+	-	-						
AR 11-10	*B. cereus s.l.*	ARGENTINA	+	+	-	-						
AR 12-1	*B. cereus s.l.*	ARGENTINA	+	+	-	-						
AR 12-2	*B. cereus s.l.*	ARGENTINA	+	+	-	-						
AR 12-3	*B. cereus s.l.*	ARGENTINA	+	+	-	-						
AR 12-4	*B. cereus s.l.*	ARGENTINA	+	+	-	-						
AR 12-5	*B. cereus s.l.*	ARGENTINA	+	-	-	-						
AR 12-6	*B. cereus s.l.*	ARGENTINA	+	+	-	-						
AR 12-7	*B. cereus s.l.*	ARGENTINA	+	+	-	-						
AR 12-8	*B. cereus s.l.*	ARGENTINA	+	+	-	+	MH618413	0.67	465	IV	CC18	this study
AR 12-9	*B. cereus s.l.*	ARGENTINA	+	+	-	+						
AR 12-10	*B. cereus s.l.*	ARGENTINA	+	+	-	-						
AR 13-1	*B. cereus s.l.*	ARGENTINA	+	+	-	-			800	V	S	Kaminska *et al.* (2015)
AR 13-1-2	*B. cereus s.l.*	ARGENTINA	+	+	-	-			655	VI	CC410-650	this study
AR 13-2	*B. cereus s.l.*	ARGENTINA	+	+	-	-						
AR 13-3	*B. cereus s.l.*	ARGENTINA	+	-	-	-						
AR 13-4	*B. cereus s.l.*	ARGENTINA	+	+	-	+						
AR 13-5	*B. cereus s.l.*	ARGENTINA	+	+	-	-						
AR 13-6	*B. cereus s.l.*	ARGENTINA	+	+	-	-						
AR 13-7	*B. cereus s.l.*	ARGENTINA	+	-	-	-						
AR 13-8	*B. cereus s.l.*	ARGENTINA	+	+	-	+						
AR 13-9	*B. cereus s.l.*	ARGENTINA	+	+	-	+	MH618414	0.70	18	IV	CC18	Kaminska *et al.* (2015)
AR 13-10	*B. cereus s.l.*	ARGENTINA	+	+	-	+						
AR 14-1	*B. cereus s.l.*	ARGENTINA	+	+	-	-						
AR 14-2	*B. cereus s.l.*	ARGENTINA	+	+	-	-						
AR 14-3	*B. cereus s.l.*	ARGENTINA	+	+	-	+	MH618415	0.70	1492	IV	CC117-1492	this study
AR 14-4	*B. cereus s.l.*	ARGENTINA	+	+	-	+						
AR 14-5	*B. cereus s.l.*	ARGENTINA	+	+	-	-			1493	II	CC614	this study
AR 14-6	*B. cereus s.l.*	ARGENTINA	+	+	-	+						
AR 14-7	*B. cereus s.l.*	ARGENTINA	+	+	-	+						
AR 14-8	*B. cereus s.l.*	ARGENTINA	+	+	-	-						
AR 14-9	*B. cereus s.l.*	ARGENTINA	+	+	-	+	MH618416	0.53	1203	IV	S	this study
AR 14-10	*B. cereus s.l.*	ARGENTINA	+	+	-	+						
AR 15-1	*B. cereus s.l.*	ARGENTINA	-	+	-	-			888	II	S	Kaminska *et al.* (2015)
AR 15-2	*B. cereus s.l.*	ARGENTINA	+	-	-	+						
AR 15-3	*B. cereus s.l.*	ARGENTINA	+	+	-	+	MH618417	0.52	225	IV	CC18	Kaminska *et al.* (2015)
AR 15-4	*B. cereus s.l.*	ARGENTINA	+	+	-	+						
AR 15-5	*B. cereus s.l.*	ARGENTINA	-	+	-	-						
AR 15-6	*B. cereus s.l.*	ARGENTINA	+	+	-	+						
AR 15-7	*B. cereus s.l.*	ARGENTINA	+	+	-	-						
AR 15-8	*B. cereus s.l.*	ARGENTINA	+	+	-	-						
AR 15-9	*B. cereus s.l.*	ARGENTINA	+	+	-	-						
AR 15-10	*B. cereus s.l.*	ARGENTINA	+	-	-	+						
AR 16-1	*B. cereus s.l.*	ARGENTINA	-	+	-	+						
AR 16-2	*B. cereus s.l.*	ARGENTINA	+	+	-	+	MH618418	0.64	117	IV	CC117-1492	this study
AR 16-3	*B. cereus s.l.*	ARGENTINA	+	+	-	+						
AR 16-4	*B. cereus s.l.*	ARGENTINA	+	+	-	+						
AR 16-5	*B. cereus s.l.*	ARGENTINA	+	+	-	+						
AR 16-6	*B. cereus s.l.*	ARGENTINA	+	+	-	+						
AR 16-7	*B. cereus s.l.*	ARGENTINA	+	+	-	-						
AR 16-8	*B. cereus s.l.*	ARGENTINA	+	+	-	-						
AR 16-9	*B. cereus s.l.*	ARGENTINA	-	+	-	+						
AR 16-10	*B. cereus s.l.*	ARGENTINA	+	+	-	+						
AR 17-1	*B. cereus s.l.*	ARGENTINA	+	+	-	+	MH618419	0.50	1515	IV	S	this study
AR 17-2	*B. cereus s.l.*	ARGENTINA	+	+	-	-						
AR 17-3	*B. cereus s.l.*	ARGENTINA	+	+	-	-						
AR 17-4	*B. cereus s.l.*	ARGENTINA	+	+	-	-						
AR 17-5	*B. cereus s.l.*	ARGENTINA	+	+	-	-						
AR 17-6	*B. cereus s.l.*	ARGENTINA	+	+	-	-						
AR 17-7	*B. cereus s.l.*	ARGENTINA	+	+	-	+						
AR 17-8	*B. cereus s.l.*	ARGENTINA	+	+	-	-						
AR 17-9	*B. cereus s.l.*	ARGENTINA	+	+	-	+						
AR 17-10	*B. cereus s.l.*	ARGENTINA	-	+	-	-						
AR 18-1	*B. cereus s.l.*	ARGENTINA	+	+	-	+						
AR 18-2	*B. cereus s.l.*	ARGENTINA	+	+	-	+	MH618420	2.14	1494	IV	CC1494	this study
AR 18-3	*B. cereus s.l.*	ARGENTINA	+	+	-	+						
AR 18-4	*B. cereus s.l.*	ARGENTINA	+	+	-	+						
AR 18-5	*B. cereus s.l.*	ARGENTINA	+	-	-	+						
AR 18-6	*B. cereus s.l.*	ARGENTINA	+	+	-	+						
AR 18-7	*B. cereus s.l.*	ARGENTINA	+	+	-	+	MH618421	0.01	866	III	S	this study
AR 18-8	*B. cereus s.l.*	ARGENTINA	+	+	-	+						
AR 18-9	*B. cereus s.l.*	ARGENTINA	+	+	-	+						
AR 18-10	*B. cereus s.l.*	ARGENTINA	-	+	-	+						
AR 19-1	*B. cereus s.l.*	ARGENTINA	+	-	-	-						
AR 19-2	*B. cereus s.l.*	ARGENTINA	+	+	-	+						
AR 19-3	*B. cereus s.l.*	ARGENTINA	+	+	-	-						
AR 19-4	*B. cereus s.l.*	ARGENTINA	+	+	-	+	MH618422	1.48	138	IV	CC18	this study
AR 19-5	*B. cereus s.l.*	ARGENTINA	+	+	-	+						
AR 19-6	*B. cereus s.l.*	ARGENTINA	+	+	-	+						
AR 19-7	*B. cereus s.l.*	ARGENTINA	+	+	-	-						
AR 19-8	*B. cereus s.l.*	ARGENTINA	+	-	-	+						
AR 19-9	*B. cereus s.l.*	ARGENTINA	+	+	-	+						
AR 19-10	*B. cereus s.l.*	ARGENTINA	+	+	-	-						
AR 20-1	*B. cereus s.l.*	ARGENTINA	+	+	-	+	MH618423	0.66	885	IV	S	this study
AR 20-2	*B. cereus s.l.*	ARGENTINA	+	+	-	+						
AR 20-3	*B. cereus s.l.*	ARGENTINA	+	+	-	+						
AR 20-4	*B. cereus s.l.*	ARGENTINA	+	+	-	-						
AR 20-5	*B. cereus s.l.*	ARGENTINA	+	+	-	+						
AR 20-6	*B. cereus s.l.*	ARGENTINA	-	+	-	-						
AR 20-7	*B. cereus s.l.*	ARGENTINA	+	+	-	-						
AR 20-8	*B. cereus s.l.*	ARGENTINA	+	+	-	+						
AR 20-9	*B. cereus s.l.*	ARGENTINA	-	+	-	-						
AR 20-10	*B. cereus s.l.*	ARGENTINA	+	+	-	-						
BF 1-1	*B. cereus s.l.*	BURKINA FASO	-	+	-	+	MH618433	0.00	1500	III	S	this study
BF 1-2	*B. cereus s.l.*	BURKINA FASO	-	+	-	+						
BF 1-3	*B. cereus s.l.*	BURKINA FASO	+	+	-	+						
BF 1-4	*B. cereus s.l.*	BURKINA FASO	-	-	-	-						

BF 1-5	*B. cereus s.l.*	BURKINA FASO	-	+	-	-						
BF 1-6	*B. cereus s.l.*	BURKINA FASO	+	+	-	+						
BF 1-7	*B. cereus s.l.*	BURKINA FASO	-	-	-	-						
BF 1-8	*B. cereus s.l.*	BURKINA FASO	+	+	-	+						
BF 1-9	*B. cereus s.l.*	BURKINA FASO	-	+	-	+						
BF 1-10	*B. cereus s.l.*	BURKINA FASO	-	+	-	+						
BF 2-1	*B. cereus s.l.*	BURKINA FASO	-	+	-	+						
BF 2-2	*B. cereus s.l.*	BURKINA FASO	-	+	-	-						
BF 2-3	*B. cereus s.l.*	BURKINA FASO	+	+	-	+	MH618434	0.81	1495	IV	S	this study
BF 2-4	*B. cereus s.l.*	BURKINA FASO	+	+	-	+						
BF 2-5	*B. cereus s.l.*	BURKINA FASO	-	+	-	+						
BF 2-6	*B. cereus s.l.*	BURKINA FASO	+	+	-	+						
BF 2-7	*B. cereus s.l.*	BURKINA FASO	-	+	-	+						
BF 2-8	*B. cereus s.l.*	BURKINA FASO	+	+	-	+						
BF 2-9	*B. cereus s.l.*	BURKINA FASO	-	+	-	-						
BF 2-10	*B. cereus s.l.*	BURKINA FASO	+	+	-	+	MH618435	0.01	1496	III	CC463-1496	this study
BF 3-1	*B. cereus s.l.*	BURKINA FASO	+	+	-	-						
BF 3-2	*B. cereus s.l.*	BURKINA FASO	+	+	-	-						
BF 3-3	*B. cereus s.l.*	BURKINA FASO	+	+	-	-						
BF 3-4	*B. cereus s.l.*	BURKINA FASO	+	+	-	-						
BF 3-5	*B. cereus s.l.*	BURKINA FASO	+	-	-	-			733	II	CC564-733	this study
BF 3-6	*B. cereus s.l.*	BURKINA FASO	+	+	-	-						
BF 3-7	*B. cereus s.l.*	BURKINA FASO	-	+	-	-						
BF 3-8	*B. cereus s.l.*	BURKINA FASO	-	-	-	-						
BF 3-9	*B. cereus s.l.*	BURKINA FASO	+	+	-	-						
BF 3-10	*B. cereus s.l.*	BURKINA FASO	-	+	-	-			696	VI	S	this study
BF 4-1	*B. cereus s.l.*	BURKINA FASO	-	+	-	+	MH618436	0.01	1497	III	S	this study
BF 4-2	*B. cereus s.l.*	BURKINA FASO	-	+	-	-						
BF 4-3	*B. cereus s.l.*	BURKINA FASO	+	+	-	+						
BF 4-4	*B. cereus s.l.*	BURKINA FASO	+	+	-	+						
BF 4-5	*B. cereus s.l.*	BURKINA FASO	+	+	-	+						
BF 4-6	*B. cereus s.l.*	BURKINA FASO	+	+	-	-						
BF 4-7	*B. cereus s.l.*	BURKINA FASO	+	+	-	+						
BF 4-8	*B. cereus s.l.*	BURKINA FASO	-	+	-	-			164	III	S	this study
BF 4-9	*B. cereus s.l.*	BURKINA FASO	+	+	-	+						
BF 4-10	*B. cereus s.l.*	BURKINA FASO	+	+	-	+						
BF 5-1	*B. cereus s.l.*	BURKINA FASO	+	+	-	+						
BF 5-2	*B. cereus s.l.*	BURKINA FASO	-	+	-	-						
BF 5-3	*B. cereus s.l.*	BURKINA FASO	-	-	-	+						
BF 5-4	*B. cereus s.l.*	BURKINA FASO	-	+	-	+						
BF 5-5	*B. cereus s.l.*	BURKINA FASO	-	+	-	+	MH618437	0.00	1501	III	S	this study
BF 5-6	*B. cereus s.l.*	BURKINA FASO	+	+	-	-						
BF 5-7	*B. cereus s.l.*	BURKINA FASO	+	+	-	+						
BF 5-8	*B. cereus s.l.*	BURKINA FASO	+	+	-	+						
BF 5-9	*B. cereus s.l.*	BURKINA FASO	-	-	-	+						
BF 5-10	*B. cereus s.l.*	BURKINA FASO	-	-	-	+						
BF 6-1	*B. cereus s.l.*	BURKINA FASO	-	-	-	+						
BF 6-2	*B. cereus s.l.*	BURKINA FASO	-	+	-	+	MH618438	0.88	1498	IV	S	this study
BF 6-3	*B. cereus s.l.*	BURKINA FASO	+	-	-	+						
BF 6-4	*B. cereus s.l.*	BURKINA FASO	-	+	-	+						
BF 6-5	*B. cereus s.l.*	BURKINA FASO	+	+	-	+	MH618439	0.69	846	IV	S	this study
BF 6-6	*B. cereus s.l.*	BURKINA FASO	-	+	-	+						
BF 6-7	*B. cereus s.l.*	BURKINA FASO	+	-	-	+						
BF 6-8	*B. cereus s.l.*	BURKINA FASO	+	+	-	+						
BF 6-9	*B. cereus s.l.*	BURKINA FASO	-	-	-	-						
BF 6-10	*B. cereus s.l.*	BURKINA FASO	+	+	-	+						
BF 7-1	*B. cereus s.l.*	BURKINA FASO	-	+	-	+	MH618440	0.01	1502	III	S	this study
BF 7-2	*B. cereus s.l.*	BURKINA FASO	-	-	-	+						
BF 7-3	*B. cereus s.l.*	BURKINA FASO	-	-	-	+						
BF 7-4	*B. cereus s.l.*	BURKINA FASO	-	+	-	+						
BF 7-5	*B. cereus s.l.*	BURKINA FASO	+	+	-	+						
BF 7-6	*B. cereus s.l.*	BURKINA FASO	-	+	-	+						
BF 7-7	*B. cereus s.l.*	BURKINA FASO	+	+	-	+						
BF 7-8	*B. cereus s.l.*	BURKINA FASO	+	+	-	+						
BF 7-9	*B. cereus s.l.*	BURKINA FASO	+	+	-	+						
BF 7-10	*B. cereus s.l.*	BURKINA FASO	+	+	-	+						
BF 8-1	*B. cereus s.l.*	BURKINA FASO	+	+	-	+	MH618441	0.98	968	IV	S	this study
BF 8-2	*B. cereus s.l.*	BURKINA FASO	+	+	-	+						
BF 8-3	*B. cereus s.l.*	BURKINA FASO	+	+	-	+						
BF 8-4	*B. cereus s.l.*	BURKINA FASO	+	-	-	-						
BF 8-5	*B. cereus s.l.*	BURKINA FASO	+	+	-	-			218	V	CC218-223	this study
BF 8-6	*B. cereus s.l.*	BURKINA FASO	+	+	-	+						
BF 8-7	*B. cereus s.l.*	BURKINA FASO	+	+	-	-						
BF 8-8	*B. cereus s.l.*	BURKINA FASO	+	-	-	+	MH618442	0.95	927	IV	CC1494	this study
BF 8-9	*B. cereus s.l.*	BURKINA FASO	+	+	-	-						
BF 8-10	*B. cereus s.l.*	BURKINA FASO	+	+	-	-			972	V	CC218-223	this study
BF 9-1	*B. cereus s.l.*	BURKINA FASO	-	+	-	-			682	II	CC614	this study
BF 9-2	*B. cereus s.l.*	BURKINA FASO	+	+	-	-						
BF 9-3	*B. cereus s.l.*	BURKINA FASO	+	+	-	+	MH618443	1.3	16	IV	CC16-798	this study
BF 9-4	*B. cereus s.l.*	BURKINA FASO	+	+	-	+						
BF 9-5	*B. cereus s.l.*	BURKINA FASO	+	+	-	+						
BF 9-6	*B. cereus s.l.*	BURKINA FASO	+	+	-	-						
BF 9-7	*B. cereus s.l.*	BURKINA FASO	+	+	-	-						
BF 9-8	*B. cereus s.l.*	BURKINA FASO	+	+	-	+						
BF 9-9	*B. cereus s.l.*	BURKINA FASO	+	+	-	+						
BF 9-10	*B. cereus s.l.*	BURKINA FASO	+	+	-	+	MH618444	0.01	463	III	CC463-1496	this study
BF 10-1	*B. cereus s.l.*	BURKINA FASO	+	+	-	-						
BF 10-2	*B. cereus s.l.*	BURKINA FASO	-	+	-	-			1503	VI	CC410-650	this study
BF 10-3	*B. cereus s.l.*	BURKINA FASO	+	+	-	-						
BF 10-4	*B. cereus s.l.*	BURKINA FASO	+	+	-	-						
BF 10-5	*B. cereus s.l.*	BURKINA FASO	+	+	-	-						
BF 10-6	*B. cereus s.l.*	BURKINA FASO	+	+	-	-			1504	II	S	this study
BF 10-7	*B. cereus s.l.*	BURKINA FASO	+	+	-	-						
BF 10-8	*B. cereus s.l.*	BURKINA FASO	+	-	-	-						
BF 10-9	*B. cereus s.l.*	BURKINA FASO	-	+	-	-						
BF 10-10	*B. cereus s.l.*	BURKINA FASO	+	+	-	-						
AY 1-1	*B. cereus s.l.*	KAZAKHSTAN	+	+	-	-						
AY 1-2	*B. cereus s.l.*	KAZAKHSTAN	+	+	-	-						
AY 1-3	*B. cereus s.l.*	KAZAKHSTAN	+	+	-	-						
AY 1-4	*B. cereus s.l.*	KAZAKHSTAN	+	+	-	-			889	II	CC889	Kaminska *et al.* (2015)
AY 1-5	*B. cereus s.l.*	KAZAKHSTAN	+	+	-	-						
AY 1-6	*B. cereus s.l.*	KAZAKHSTAN	-	-	-	-						
AY 1-7	*B. cereus s.l.*	KAZAKHSTAN	+	+	-	+	MH618424	0.53	12	IV	S	this study
AY 1-8	*B. cereus s.l.*	KAZAKHSTAN	+	+	-	+						
AY 1-9	*B. cereus s.l.*	KAZAKHSTAN	+	+	-	-						

AY 1-10	*B. cereus s.l.*	KAZAKHSTAN	+	+	-	+	MH618425	0.01	1505	III	S	this study
AY 2-1	*B. cereus s.l.*	KAZAKHSTAN	+	+	-	+		0.55	12	IV	S	this study
AY 2-2	*B. cereus s.l.*	KAZAKHSTAN	+	+	-	-			889	II	CC889	Kaminska *et al.* (2015)
AY 2-3	*B. cereus s.l.*	KAZAKHSTAN	-	-	-	-			889	II	CC889	Kaminska *et al.* (2015)
AY 2-4	*B. cereus s.l.*	KAZAKHSTAN	+	+	-	+						
AY 2-5	*B. cereus s.l.*	KAZAKHSTAN	+	+	-	-						
AY 2-6	*B. cereus s.l.*	KAZAKHSTAN	+	+	-	-						
AY 2-7	*B. cereus s.l.*	KAZAKHSTAN	+	+	-	+						
AY 2-8	*B. cereus s.l.*	KAZAKHSTAN	+	+	-	+						
AY 2-9	*B. cereus s.l.*	KAZAKHSTAN	+	+	-	+						
AY 2-10	*B. cereus s.l.*	KAZAKHSTAN	+	+	-	-						
AY 3-1	*B. cereus s.l.*	KAZAKHSTAN	+	+	-	+	MH618426	0.00	813	II	S	Kaminska *et al.* (2015)
AY 3-2	*B. cereus s.l.*	KAZAKHSTAN	+	+	-	+						
AY 3-3	*B. cereus s.l.*	KAZAKHSTAN	+	+	-	-			889	II	CC889	Kaminska *et al.* (2015)
AY 3-4	*B. cereus s.l.*	KAZAKHSTAN	+	+	-	+						
AY 3-5	*B. cereus s.l.*	KAZAKHSTAN	+	+	-	-						
AY 3-6	*B. cereus s.l.*	KAZAKHSTAN	+	+	-	+						
AY 3-7	*B. cereus s.l.*	KAZAKHSTAN	+	+	-	+						
AY 3-8	*B. cereus s.l.*	KAZAKHSTAN	+	+	-	-						
AY 3-9	*B. cereus s.l.*	KAZAKHSTAN	+	+	-	-						
AY 3-10	*B. cereus s.l.*	KAZAKHSTAN	+	+	-	-						
AY 4-1	*B. cereus s.l.*	KAZAKHSTAN	+	+	-	+	MH618427	0.83	797	IV	S	Kaminska *et al.* (2015)
AY 4-2	*B. cereus s.l.*	KAZAKHSTAN	+	-	-	-						
AY 4-3	*B. cereus s.l.*	KAZAKHSTAN	+	+	-	-						
AY 4-4	*B. cereus s.l.*	KAZAKHSTAN	-	+	-	-						
AY 4-5	*B. cereus s.l.*	KAZAKHSTAN	+	+	-	-						
AY 4-6	*B. cereus s.l.*	KAZAKHSTAN	-	+	-	-						
AY 4-7	*B. cereus s.l.*	KAZAKHSTAN	+	+	-	-						
AY 4-8	*B. cereus s.l.*	KAZAKHSTAN	+	-	-	-			890	II	S	Kaminska *et al.* (2015)
AY 4-9	*B. cereus s.l.*	KAZAKHSTAN	+	+	-	+						
AY 4-10	*B. cereus s.l.*	KAZAKHSTAN	+	+	-	-						
AY 5-1	*B. cereus s.l.*	KAZAKHSTAN	-	+	-	+			294	II	CC294	Kaminska *et al.* (2015)
AY 5-2	*B. cereus s.l.*	KAZAKHSTAN	+	+	-	-			889	II	CC889	Kaminska *et al.* (2015)
AY 5-3	*B. cereus s.l.*	KAZAKHSTAN	+	+	-	+		0.00	460	III	S	Kaminska *et al.* (2015)
AY 5-4	*B. cereus s.l.*	KAZAKHSTAN	+	+	-	-						
AY 5-5	*B. cereus s.l.*	KAZAKHSTAN	+	+	-	-						
AY 5-6	*B. cereus s.l.*	KAZAKHSTAN	+	+	-	-			891	II	S	Kaminska *et al.* (2015)
AY 5-7	*B. cereus s.l.*	KAZAKHSTAN	+	+	-	-			892	II	S	Kaminska *et al.* (2015)
AY 5-8	*B. cereus s.l.*	KAZAKHSTAN	-	+	-	-			893	VI	S	Kaminska *et al.* (2015)
AY 5-9	*B. cereus s.l.*	KAZAKHSTAN	+	+	-	-			894	II	S	Kaminska *et al.* (2015)
AY 5-10	*B. cereus s.l.*	KAZAKHSTAN	+	+	-	-			895	II	S	Kaminska *et al.* (2015)
AY 6-1	*B. cereus s.l.*	KAZAKHSTAN	+	+	-	-			889	II	CC889	Kaminska *et al.* (2015)
AY 6-2	*B. cereus s.l.*	KAZAKHSTAN	+	-	-	-			889	II	CC889	Kaminska *et al.* (2015)
AY 6-3	*B. cereus s.l.*	KAZAKHSTAN	+	+	-	-			889	II	CC889	Kaminska *et al.* (2015)
AY 6-4	*B. cereus s.l.*	KAZAKHSTAN	+	+	-	-			896	II	CC889	Kaminska *et al.* (2015)
AY 6-5	*B. cereus s.l.*	KAZAKHSTAN	+	-	-	-			814	VI	S	Kaminska *et al.* (2015)
AY 6-6	*B. cereus s.l.*	KAZAKHSTAN	+	+	-	-						
AY 6-7	*B. cereus s.l.*	KAZAKHSTAN	+	+	-	-						
AY 6-8	*B. cereus s.l.*	KAZAKHSTAN	+	+	-	+						
AY 6-9	*B. cereus s.l.*	KAZAKHSTAN	+	+	-	-						
AY 6-10	*B. cereus s.l.*	KAZAKHSTAN	+	+	-	-						
AY 7-1	*B. cereus s.l.*	KAZAKHSTAN	+	+	-	-			798	IV	CC16-798	Kaminska *et al.* (2015)
AY 7-2	*B. cereus s.l.*	KAZAKHSTAN	+	+	-	-			897	VI	S	Kaminska *et al.* (2015)
AY 7-3	*B. cereus s.l.*	KAZAKHSTAN	-	+	-	-						
AY 7-4	*B. cereus s.l.*	KAZAKHSTAN	+	+	-	-						
AY 7-5	*B. cereus s.l.*	KAZAKHSTAN	+	+	-	+						
AY 7-6	*B. cereus s.l.*	KAZAKHSTAN	+	+	-	-						
AY 7-7	*B. cereus s.l.*	KAZAKHSTAN	+	+	-	-						
AY 7-8	*B. cereus s.l.*	KAZAKHSTAN	-	-	-	-			897	VI	S	Kaminska *et al.* (2015)
AY 7-9	*B. cereus s.l.*	KAZAKHSTAN	-	+	-	-						
AY 7-10	*B. cereus s.l.*	KAZAKHSTAN	+	+	-	-						
AY 8-1	*B. cereus s.l.*	KAZAKHSTAN	+	+	-	+	MH618428	0.86	797	IV	S	Kaminska *et al.* (2015)
AY 8-2	*B. cereus s.l.*	KAZAKHSTAN	+	+	-	+						
AY 8-3	*B. cereus s.l.*	KAZAKHSTAN	+	+	-	+						
AY 8-4	*B. cereus s.l.*	KAZAKHSTAN	+	+	-	+						
AY 8-5	*B. cereus s.l.*	KAZAKHSTAN	-	+	-	-						
AY 8-6	*B. cereus s.l.*	KAZAKHSTAN	+	+	-	-						
AY 8-7	*B. cereus s.l.*	KAZAKHSTAN	+	+	-	-						
AY 8-8	*B. cereus s.l.*	KAZAKHSTAN	+	-	-	-						
AY 8-9	*B. cereus s.l.*	KAZAKHSTAN	+	+	-	-						
AY 8-10	*B. cereus s.l.*	KAZAKHSTAN	+	+	-	-						
AY 9-1	*B. cereus s.l.*	KAZAKHSTAN	+	+	-	+			815	II	CC889	Kaminska *et al.* (2015)
AY 9-2	*B. cereus s.l.*	KAZAKHSTAN	+	+	-	+						
AY 9-3	*B. cereus s.l.*	KAZAKHSTAN	+	+	-	+						
AY 9-4	*B. cereus s.l.*	KAZAKHSTAN	-	-	-	-			898	II	CC294	Kaminska *et al.* (2015)
AY 9-5	*B. cereus s.l.*	KAZAKHSTAN	+	+	-	-						
AY 9-6	*B. cereus s.l.*	KAZAKHSTAN	+	+	-	-			889	II	CC889	Kaminska *et al.* (2015)
AY 9-7	*B. cereus s.l.*	KAZAKHSTAN	+	+	-	-						
AY 9-8	*B. cereus s.l.*	KAZAKHSTAN	+	-	-	-						
AY 9-9	*B. cereus s.l.*	KAZAKHSTAN	+	+	-	-						
AY 9-10	*B. cereus s.l.*	KAZAKHSTAN	+	-	-	-			889	II	CC889	Kaminska *et al.* (2015)
AY 10-1	*B. cereus s.l.*	KAZAKHSTAN	+	-	-	-			816	II	S	Kaminska *et al.* (2015)
AY 10-2	*B. cereus s.l.*	KAZAKHSTAN	-	+	-	-						
AY 10-3	*B. cereus s.l.*	KAZAKHSTAN	+	+	-	-						
AY 10-4	*B. cereus s.l.*	KAZAKHSTAN	-	+	-	-						
AY 10-5	*B. cereus s.l.*	KAZAKHSTAN	+	-	-	-			899	II	S	Kaminska *et al.* (2015)
AY 10-6	*B. cereus s.l.*	KAZAKHSTAN	+	+	-	-						
AY 10-7	*B. cereus s.l.*	KAZAKHSTAN	+	+	-	-						
AY 10-8	*B. cereus s.l.*	KAZAKHSTAN	+	+	-	-						
AY 10-9	*B. cereus s.l.*	KAZAKHSTAN	+	-	-	-						
AY 10-10	*B. cereus s.l.*	KAZAKHSTAN	+	+	-	+						
AY 11-1	*B. cereus s.l.*	KAZAKHSTAN	+	-	-	-			889	II	CC889	Kaminska *et al.* (2015)
AY 11-2	*B. cereus s.l.*	KAZAKHSTAN	+	+	-	-						
AY 11-3	*B. cereus s.l.*	KAZAKHSTAN	+	+	-	+						
AY 11-4	*B. cereus s.l.*	KAZAKHSTAN	+	+	-	+						
AY 11-5	*B. cereus s.l.*	KAZAKHSTAN	+	+	-	-						
AY 11-6	*B. cereus s.l.*	KAZAKHSTAN	+	+	-	+						
AY 11-7	*B. cereus s.l.*	KAZAKHSTAN	+	+	-	-						
AY 11-8	*B. cereus s.l.*	KAZAKHSTAN	+	+	-	+						
AY 11-9	*B. cereus s.l.*	KAZAKHSTAN	+	+	-	+	MH618429	0.47	1506	IV	S	this study
AY 11-10	*B. cereus s.l.*	KAZAKHSTAN	+	+	-	-						
AY 12-1	*B. cereus s.l.*	KAZAKHSTAN	+	+	-	+						
AY 12-2	*B. cereus s.l.*	KAZAKHSTAN	+	+	-	+						
AY 12-3	*B. cereus s.l.*	KAZAKHSTAN	-	+	-	-						
AY 12-4	*B. cereus s.l.*	KAZAKHSTAN	+	+	-	-						

AY 12-5	*B. cereus s.l.*	KAZAKHSTAN	+	+	-	-						
AY 12-6	*B. cereus s.l.*	KAZAKHSTAN	+	+	-	-						
AY 12-7	*B. cereus s.l.*	KAZAKHSTAN	+	+	-	-						
AY 12-8	*B. cereus s.l.*	KAZAKHSTAN	+	+	-	+						
AY 12-9	*B. cereus s.l.*	KAZAKHSTAN	+	+	-	+						
AY 12-10	*B. cereus s.l.*	KAZAKHSTAN	+	+	-	+						
AY 13-1	*B. cereus s.l.*	KAZAKHSTAN	+	+	-	-						
AY 13-2	*B. cereus s.l.*	KAZAKHSTAN	+	+	-	+						
AY 13-3	*B. cereus s.l.*	KAZAKHSTAN	+	+	-	+						
AY 13-4	*B. cereus s.l.*	KAZAKHSTAN	+	+	-	-			889	II	CC889	Kaminska *et al.* (2015)
AY 13-5	*B. cereus s.l.*	KAZAKHSTAN	+	+	-	-						
AY 13-6	*B. cereus s.l.*	KAZAKHSTAN	+	+	-	-			900	II	S	Kaminska *et al.* (2015)
AY 13-7	*B. cereus s.l.*	KAZAKHSTAN	+	+	-	-			889	II	CC889	Kaminska *et al.* (2015)
AY 13-8	*B. cereus s.l.*	KAZAKHSTAN	+	+	-	-						
AY 13-9	*B. cereus s.l.*	KAZAKHSTAN	+	+	-	+	MH618430	0.80	1516	IV	S	this study
AY 13-10	*B. cereus s.l.*	KAZAKHSTAN	+	+	-	-						
AY 14-1	*B. cereus s.l.*	KAZAKHSTAN	+	+	-	-						
AY 14-2	*B. cereus s.l.*	KAZAKHSTAN	+	+	-	-						
AY 14-3	*B. cereus s.l.*	KAZAKHSTAN	+	+	-	-						
AY 14-4	*B. cereus s.l.*	KAZAKHSTAN	-	+	-	+						
AY 14-5	*B. cereus s.l.*	KAZAKHSTAN	+	+	-	-						
AY 14-6	*B. cereus s.l.*	KAZAKHSTAN	+	+	-	-						
AY 14-7	*B. cereus s.l.*	KAZAKHSTAN	-	+	-	-						
AY 14-8	*B. cereus s.l.*	KAZAKHSTAN	+	+	-	-						
AY 14-9	*B. cereus s.l.*	KAZAKHSTAN	+	+	-	-			901	II	CC294	Kaminska *et al.* (2015)
AY 14-10	*B. cereus s.l.*	KAZAKHSTAN	+	+	-	-						
AY 15-1	*B. cereus s.l.*	KAZAKHSTAN	+	+	-	-						
AY 15-2	*B. cereus s.l.*	KAZAKHSTAN	+	+	-	-			902	II	CC889	Kaminska *et al.* (2015)
AY 15-3	*B. cereus s.l.*	KAZAKHSTAN	+	+	-	-						
AY 15-4	*B. cereus s.l.*	KAZAKHSTAN	+	+	-	-						
AY 15-5	*B. cereus s.l.*	KAZAKHSTAN	-	+	-	-						
AY 15-6	*B. cereus s.l.*	KAZAKHSTAN	+	-	-	-			889	II	CC889	Kaminska *et al.* (2015)
AY 15-7	*B. cereus s.l.*	KAZAKHSTAN	+	+	-	-			901	II	CC294	Kaminska *et al.* (2015)
AY 15-8	*B. cereus s.l.*	KAZAKHSTAN	+	+	-	+			903	IV	S	Kaminska *et al.* (2015)
AY 15-9	*B. cereus s.l.*	KAZAKHSTAN	+	+	-	-						
AY 15-10	*B. cereus s.l.*	KAZAKHSTAN	+	+	-	-						
AY 16-1	*B. cereus s.l.*	KAZAKHSTAN	+	+	-	-						
AY 16-2	*B. cereus s.l.*	KAZAKHSTAN	+	+	-	-						
AY 16-3	*B. cereus s.l.*	KAZAKHSTAN	+	+	-	+						
AY 16-4	*B. cereus s.l.*	KAZAKHSTAN	+	+	-	-						
AY 16-5	*B. cereus s.l.*	KAZAKHSTAN	-	+	-	-						
AY 16-6	*B. cereus s.l.*	KAZAKHSTAN	+	-	-	-			900	II	S	Kaminska *et al.* (2015)
AY 16-7	*B. cereus s.l.*	KAZAKHSTAN	+	+	-	-						
AY 16-8	*B. cereus s.l.*	KAZAKHSTAN	+	+	-	+						
AY 16-9	*B. cereus s.l.*	KAZAKHSTAN	+	+	-	+						
AY 16-10	*B. cereus s.l.*	KAZAKHSTAN	+	+	-	-						
AY 17-1	*B. cereus s.l.*	KAZAKHSTAN	-	+	-	-						
AY 17-2	*B. cereus s.l.*	KAZAKHSTAN	-	+	-	-						
AY 17-3	*B. cereus s.l.*	KAZAKHSTAN	+	+	-	-						
AY 17-4	*B. cereus s.l.*	KAZAKHSTAN	+	+	-	-						
AY 17-5	*B. cereus s.l.*	KAZAKHSTAN	+	+	-	+						
AY 17-6	*B. cereus s.l.*	KAZAKHSTAN	-	-	-	-						
AY 17-7	*B. cereus s.l.*	KAZAKHSTAN	+	+	-	-			889	II	CC889	Kaminska *et al.* (2015)
AY 17-8	*B. cereus s.l.*	KAZAKHSTAN	+	+	-	-						
AY 17-9	*B. cereus s.l.*	KAZAKHSTAN	+	+	-	-						
AY 17-10	*B. cereus s.l.*	KAZAKHSTAN	+	+	-	-						
AY 18-1	*B. cereus s.l.*	KAZAKHSTAN	+	+	-	-						
AY 18-2	*B. cereus s.l.*	KAZAKHSTAN	+	+	-	+	MH618431	0.00	1518	II	CC1517-1518	this study
AY 18-3	*B. cereus s.l.*	KAZAKHSTAN	+	+	-	-			889	II	CC889	Kaminska *et al.* (2015)
AY 18-4	*B. cereus s.l.*	KAZAKHSTAN	+	+	-	-			889	II	CC889	Kaminska *et al.* (2015)
AY 18-5	*B. cereus s.l.*	KAZAKHSTAN	+	+	-	-						
AY 18-6	*B. cereus s.l.*	KAZAKHSTAN	-	+	-	-						
AY 18-7	*B. cereus s.l.*	KAZAKHSTAN	+	+	-	+						
AY 18-8	*B. cereus s.l.*	KAZAKHSTAN	+	+	-	-						
AY 18-9	*B. cereus s.l.*	KAZAKHSTAN	+	+	-	-						
AY 18-10	*B. cereus s.l.*	KAZAKHSTAN	+	-	-	-						
AY 19-1	*B. cereus s.l.*	KAZAKHSTAN	+	+	-	+						
AY 19-2	*B. cereus s.l.*	KAZAKHSTAN	+	-	-	-						
AY 19-3	*B. cereus s.l.*	KAZAKHSTAN	+	+	-	-						
AY 19-4	*B. cereus s.l.*	KAZAKHSTAN	+	+	-	-						
AY 19-5	*B. cereus s.l.*	KAZAKHSTAN	+	+	-	-			889	II	CC889	Kaminska *et al.* (2015)
AY 19-6	*B. cereus s.l.*	KAZAKHSTAN	+	+	-	-			900	II	S	Kaminska *et al.* (2015)
AY 19-7	*B. cereus s.l.*	KAZAKHSTAN	+	+	-	-						
AY 19-8	*B. cereus s.l.*	KAZAKHSTAN	+	+	-	-						
AY 19-9	*B. cereus s.l.*	KAZAKHSTAN	+	+	-	+						
AY 19-10	*B. cereus s.l.*	KAZAKHSTAN	+	+	-	+	MH618432	0.00	1517	II	1517-1518	this study
AY 20-1	*B. cereus s.l.*	KAZAKHSTAN	+	+	-	+						
AY 20-2	*B. cereus s.l.*	KAZAKHSTAN	+	+	-	+						
AY 20-3	*B. cereus s.l.*	KAZAKHSTAN	+	+	-	-			889	II	CC889	Kaminska *et al.* (2015)
AY 20-4	*B. cereus s.l.*	KAZAKHSTAN	+	+	-	-						
AY 20-5	*B. cereus s.l.*	KAZAKHSTAN	+	+	-	-						
AY 20-6	*B. cereus s.l.*	KAZAKHSTAN	+	+	-	-						
AY 20-7	*B. cereus s.l.*	KAZAKHSTAN	+	+	-	+						
AY 20-8	*B. cereus s.l.*	KAZAKHSTAN	+	+	-	+		0.56	12	IV	S	this study
AY 20-9	*B. cereus s.l.*	KAZAKHSTAN	+	+	-	-						
AY 20-10	*B. cereus s.l.*	KAZAKHSTAN	+	+	-	+						
TE 1-1	*B. cereus s.l.*	KENYA	-	+	-	+	MH618469	0.01	842	III	S	Kaminska *et al.* (2015)
TE 1-3	*B. cereus s.l.*	KENYA	+	+	-	+						
TE 1-4	*B. cereus s.l.*	KENYA	-	+	-	+						
TE 1-5	*B. cereus s.l.*	KENYA	+	+	-	+						
TE 1-6	*B. cereus s.l.*	KENYA	+	+	-	+						
TE 1-7	*B. cereus s.l.*	KENYA	-	+	-	+						
TE 1-8	*B. cereus s.l.*	KENYA	+	+	-	+						
TE 1-9	*B. cereus s.l.*	KENYA	+	+	-	+	MH618470	1.31	785	IV	S	Kaminska *et al.* (2015)
TE 1-10	*B. cereus s.l.*	KENYA	+	+	-	+						
TE 1-11	*B. cereus s.l.*	KENYA	+	+	-	+						
TE 1-13	*B. cereus s.l.*	KENYA	+	+	-	+						
TE 2-1	*B. cereus s.l.*	KENYA	+	+	-	+	MH618471	0.62	799	IV	S	Kaminska *et al.* (2015)
TE 2-2	*B. cereus s.l.*	KENYA	-	+	-	+						
TE 2-3	*B. cereus s.l.*	KENYA	+	+	-	+						
TE 2-4	*B. cereus s.l.*	KENYA	+	+	-	+						
TE 2-5	*B. cereus s.l.*	KENYA	+	+	-	+						
TE 2-6	*B. cereus s.l.*	KENYA	-	+	-	+						
TE 2-7	*B. cereus s.l.*	KENYA	-	-	-	-						
TE 2-8	*B. cereus s.l.*	KENYA	+	+	-	+						

TE 2-9	*B. cereus s.l.*	KENYA	+	+	-	+						
TE 2-12	*B. cereus s.l.*	KENYA	+	+	-	+	MH618472	0.00	876	III	CC1512	Kaminska *et al.* (2015)
TE 2-13	*B. cereus s.l.*	KENYA	+	+	-	+	MH618473	1.13	877	IV	S	Kaminska *et al.* (2015)
TE 3-1	*B. cereus s.l.*	KENYA	+	+	-	+		0.90	801	IV	S	Kaminska *et al.* (2015)
TE 3-2	*B. cereus s.l.*	KENYA	+	+	-	-						
TE 3-3	*B. cereus s.l.*	KENYA	+	+	-	+						
TE 3-4	*B. cereus s.l.*	KENYA	-	+	-	+						
TE 3-5	*B. cereus s.l.*	KENYA	+	+	-	+						
TE 3-6	*B. cereus s.l.*	KENYA	-	+	-	+						
TE 3-7	*B. cereus s.l.*	KENYA	-	+	-	+						
TE 3-8	*B. cereus s.l.*	KENYA	+	+	-	+	MH618474	0.01	863	III	S	Kaminska *et al.* (2015)
TE 3-9	*B. cereus s.l.*	KENYA	+	+	-	+						
TE 3-10	*B. cereus s.l.*	KENYA	-	+	-	+						
TE 3-12	*B. cereus s.l.*	KENYA	-	+	-	+						
TE 4-1	*B. cereus s.l.*	KENYA	-	+	-	+	MH618475	0.01	802	III	S	Kaminska *et al.* (2015)
TE 4-2	*B. cereus s.l.*	KENYA	+	+	-	+	MH618476	0.00	864	IV	CC4-864	Kaminska *et al.* (2015)
TE 4-3	*B. cereus s.l.*	KENYA	-	+	-	+						
TE 4-4	*B. cereus s.l.*	KENYA	+	+	-	+						
TE 4-5	*B. cereus s.l.*	KENYA	+	+	-	+						
TE 4-6	*B. cereus s.l.*	KENYA	+	+	-	+						
TE 4-7	*B. cereus s.l.*	KENYA	+	+	-	+						
TE 4-8	*B. cereus s.l.*	KENYA	-	+	-	+						
TE 4-9	*B. cereus s.l.*	KENYA	+	+	-	+						
TE 4-10	*B. cereus s.l.*	KENYA	-	+	-	+						
TE 4-11	*B. cereus s.l.*	KENYA	-	+	-	+						
TE 5-1	*B. cereus s.l.*	KENYA	-	+	-	+		1.01	865	III	S	Kaminska *et al.* (2015)
TE 5-2	*B. cereus s.l.*	KENYA	+	+	-	+	MH618477	2.52	803	IV	S	Kaminska *et al.* (2015)
TE 5-3	*B. cereus s.l.*	KENYA	-	+	-	+						
TE 5-4	*B. cereus s.l.*	KENYA	-	+	-	+						
TE 5-5	*B. cereus s.l.*	KENYA	-	+	-	+						
TE 5-6	*B. cereus s.l.*	KENYA	-	+	-	+						
TE 5-7	*B. cereus s.l.*	KENYA	-	+	-	+						
TE 5-8	*B. cereus s.l.*	KENYA	-	+	-	+						
TE 5-9	*B. cereus s.l.*	KENYA	-	+	-	+						
TE 5-10	*B. cereus s.l.*	KENYA	+	+	-	+						
TE 6-1	*B. cereus s.l.*	KENYA	+	+	-	+	MH618478	1.77	804	IV	S	Kaminska *et al.* (2015)
TE 6-2	*B. cereus s.l.*	KENYA	-	+	-	+						
TE 6-3	*B. cereus s.l.*	KENYA	+	+	-	+						
TE 6-4	*B. cereus s.l.*	KENYA	+	+	-	+						
TE 6-5	*B. cereus s.l.*	KENYA	+	+	-	+						
TE 6-6	*B. cereus s.l.*	KENYA	+	+	-	+						
TE 6-7	*B. cereus s.l.*	KENYA	-	+	-	+	MH618479	0.01	866	III	S	Kaminska *et al.* (2015)
TE 6-8	*B. cereus s.l.*	KENYA	+	+	-	+	MH618480	1.59	867	IV	S	Kaminska *et al.* (2015)
TE 6-9	*B. cereus s.l.*	KENYA	-	+	-	+						
TE 6-10	*B. cereus s.l.*	KENYA	-	+	-	+						
TE 6-12	*B. cereus s.l.*	KENYA	+	+	-	+						
TE 6-13	*B. cereus s.l.*	KENYA	+	+	-	+						
TE 7-1	*B. cereus s.l.*	KENYA	+	-	-	+	MH618481	0.90	822	IV	S	Kaminska *et al.* (2015)
TE 7-2	*B. cereus s.l.*	KENYA	-	+	-	+						
TE 7-3	*B. cereus s.l.*	KENYA	-	+	-	+						
TE 7-4	*B. cereus s.l.*	KENYA	-	+	-	+						
TE 7-5	*B. cereus s.l.*	KENYA	+	+	-	+						
TE 7-6	*B. cereus s.l.*	KENYA	+	+	-	+						
TE 7-7	*B. cereus s.l.*	KENYA	+	+	-	+	MH618482	0.00	868	III	S	Kaminska *et al.* (2015)
TE 7-8	*B. cereus s.l.*	KENYA	+	+	-	+						
TE 7-9	*B. cereus s.l.*	KENYA	-	+	-	+						
TE 7-10	*B. cereus s.l.*	KENYA	-	+	-	+						
TE 8-1	*B. cereus s.l.*	KENYA	+	+	-	+	MH618483	0.92	870	IV	S	Kaminska *et al.* (2015)
TE 8-2	*B. cereus s.l.*	KENYA	+	+	-	+			871	IV	S	Kaminska *et al.* (2015)
TE 8-3	*B. cereus s.l.*	KENYA	+	+	-	+	MH618484	0.54	872	IV	S	Kaminska *et al.* (2015)
TE 8-4	*B. cereus s.l.*	KENYA	+	+	-	+			805	IV	S	Kaminska *et al.* (2015)
TE 8-5	*B. cereus s.l.*	KENYA	+	+	-	+						
TE 8-6	*B. cereus s.l.*	KENYA	-	+		-			873	III	S	Kaminska *et al.* (2015)
TE 8-7	*B. cereus s.l.*	KENYA	+	+	-	+						
TE 8-8	*B. cereus s.l.*	KENYA	+	+	-	+	MH618485	1.55	874	IV	CC810-874	Kaminska *et al.* (2015)
TE 8-9	*B. cereus s.l.*	KENYA	+	+	-	+						
TE 8-10	*B. cereus s.l.*	KENYA	+	+	-	+						
TE 8-12	*B. cereus s.l.*	KENYA	+	+	-	+						
TE 9-1	*B. cereus s.l.*	KENYA	-	+	-	+	MH618486	0.04	823	III	S	Kaminska *et al.* (2015)
TE 9-2	*B. cereus s.l.*	KENYA	+	+	-	+						
TE 9-3	*B. cereus s.l.*	KENYA	-	-	-	-						
TE 9-4	*B. cereus s.l.*	KENYA	+	+	-	+						
TE 9-5	*B. cereus s.l.*	KENYA	+	+	-	+						
TE 9-6	*B. cereus s.l.*	KENYA	-	-	-	+						
TE 9-7	*B. cereus s.l.*	KENYA	+	+	-	+						
TE 9-8	*B. cereus s.l.*	KENYA	+	+	-	+						
TE 9-9	*B. cereus s.l.*	KENYA	+	+	-	+						
TE 9-10	*B. cereus s.l.*	KENYA	+	+	-	+						
TE 9-11	*B. cereus s.l.*	KENYA	+	+	-	+						
TE 9-12	*B. cereus s.l.*	KENYA	-	+	-	+						
TE 10-1	*B. cereus s.l.*	KENYA	+	+	-	+	MH618487	0.80	806	IV	S	Kaminska *et al.* (2015)
TE 10-2	*B. cereus s.l.*	KENYA	+	+	-	+						
TE 10-3	*B. cereus s.l.*	KENYA	+	+	-	+						
TE 10-4	*B. cereus s.l.*	KENYA	-	+	-	+						
TE 10-5	*B. cereus s.l.*	KENYA	+	+	-	+						
TE 10-6	*B. cereus s.l.*	KENYA	+	+	-	+						
TE 10-7	*B. cereus s.l.*	KENYA	-	+	-	+						
TE 10-8	*B. cereus s.l.*	KENYA	+	+	-	+						
TE 10-9	*B. cereus s.l.*	KENYA	-	+	-	-						
TE 10-10	*B. cereus s.l.*	KENYA	-	+	-	+						
SH 1-1	*B. cereus s.l.*	KENYA	+	+	-	+	MH618461	0.78	824	IV	S	Kaminska *et al.* (2015)
SH-1-2	*B. cereus s.l.*	KENYA	+	+	-	+						
SH 1-3	*B. cereus s.l.*	KENYA	+	+	-	+						
SH 1-4	*B. cereus s.l.*	KENYA	+	+	-	+						
SH 1-5	*B. cereus s.l.*	KENYA	+	+	-	+						
SH 1-6	*B. cereus s.l.*	KENYA	+	+	-	+						
SH 1-7	*B. cereus s.l.*	KENYA	+	+	-	+						
SH 1-8	*B. cereus s.l.*	KENYA	+	+	-	+						
SH 1-9	*B. cereus s.l.*	KENYA	+	+	-	+						
SH 1-10	*B. cereus s.l.*	KENYA	-	+	-	-						
SH 2-1	*B. cereus s.l.*	KENYA	+	+	-	+			879	IV	CC879-884	Kaminska *et al.* (2015)
SH 2-2	*B. cereus s.l.*	KENYA	+	+	-	+						
SH 2-3	*B. cereus s.l.*	KENYA	+	+	-	+			807	IV	S	Kaminska *et al.* (2015)
SH 2-4	*B. cereus s.l.*	KENYA	-	+	-	-						
SH 2-5	*B. cereus s.l.*	KENYA	+	+	-	+						

SH 2-6	*B. cereus s.l.*	KENYA	+	+	-	+						
SH 2-7	*B. cereus s.l.*	KENYA	-	+	-	-						
SH 2-8	*B. cereus s.l.*	KENYA	+	+	-	+						
SH 2-9	*B. cereus s.l.*	KENYA	-	+	-	+						
SH 2-10	*B. cereus s.l.*	KENYA	+	+	-	+						
SH 3-1	*B. cereus s.l.*	KENYA	+	+	-	+	MH618462	0.55	808	IV	S	Kaminska *et al.* (2015)
SH 3-2	*B. cereus s.l.*	KENYA	+	+	-	+						
SH 3-3	*B. cereus s.l.*	KENYA	+	+	-	+						
SH 3-4	*B. cereus s.l.*	KENYA	+	+	-	+						
SH 3-5	*B. cereus s.l.*	KENYA	+	+	-	+						
SH 3-6	*B. cereus s.l.*	KENYA	+	+	-	+						
SH 3-7	*B. cereus s.l.*	KENYA	+	+	-	+						
SH 3-8	*B. cereus s.l.*	KENYA	+	+	-	+						
SH 3-9	*B. cereus s.l.*	KENYA	+	+	-	+						
SH 3-10	*B. cereus s.l.*	KENYA	-	+	-	+						
SH 4-1	*B. cereus s.l.*	KENYA	-	+	-	-			825	VIII	CC764-825	Kaminska *et al.* (2015)
SH 4-2	*B. cereus s.l.*	KENYA	-	+	-	+						
SH 4-3	*B. cereus s.l.*	KENYA	+	+	-	+						
SH 4-4	*B. cereus s.l.*	KENYA	+	+	-	-						
SH 4-5	*B. cereus s.l.*	KENYA	-	-	-	-						
SH 4-6	*B. cereus s.l.*	KENYA	-	-	-	+						
SH 4-7	*B. cereus s.l.*	KENYA	-	+	-	+						
SH 4-8	*B. cereus s.l.*	KENYA	-	+	-	+						
SH 4-9	*B. cereus s.l.*	KENYA	+	+	-	+						
SH 4-10	*B. cereus s.l.*	KENYA	-	+	-	+						
SH 5-1	*B. cereus s.l.*	KENYA	+	+	-	+			809	IV	S	Kaminska *et al.* (2015)
SH 5-2	*B. cereus s.l.*	KENYA	-	+	-	-			880	VIII	S	Kaminska *et al.* (2015)
SH 5-3	*B. cereus s.l.*	KENYA	+	+	-	+	MH618463	1.24	881	IV	S	Kaminska *et al.* (2015)
SH 5-4	*B. cereus s.l.*	KENYA	-	+	-	-			1566	VIII	CC1566-1567	this study
SH 5-5	*B. cereus s.l.*	KENYA	-	+	-	+						
SH 5-6	*B. cereus s.l.*	KENYA	+	+	-	+						
SH 5-7	*B. cereus s.l.*	KENYA	+	+	-	+						
SH 5-8	*B. cereus s.l.*	KENYA	+	+	-	+						
SH 5-9	*B. cereus s.l.*	KENYA	+	+	-	+						
SH 5-10	*B. cereus s.l.*	KENYA	+	+	-	+	MH618464	0.69	882	IV	S	Kaminska *et al.* (2015)
SH 6-1	*B. cereus s.l.*	KENYA	+	+	-	+			810	IV	CC810-874	Kaminska *et al.* (2015)
SH 6-2	*B. cereus s.l.*	KENYA	+	+	-	+						
SH 6-3	*B. cereus s.l.*	KENYA	-	-	-	+						
SH 6-4	*B. cereus s.l.*	KENYA	+	+	-	+						
SH 6-5	*B. cereus s.l.*	KENYA	+	+	-	+						
SH 6-6	*B. cereus s.l.*	KENYA	+	+	-	+			883	IV	S	Kaminska *et al.* (2015)
SH 6-7	*B. cereus s.l.*	KENYA	+	+	-	+						
SH 6-8	*B. cereus s.l.*	KENYA	+	+	-	+						
SH 6-9	*B. cereus s.l.*	KENYA	+	+	-	+						
SH 6-10	*B. cereus s.l.*	KENYA	+	+	-	+						
SH 7-1	*B. cereus s.l.*	KENYA	+	+	-	+	MH618465	0.00	826	II	S	Kaminska *et al.* (2015)
SH 7-2	*B. cereus s.l.*	KENYA	-	-	-	+						
SH 7-3	*B. cereus s.l.*	KENYA	+	+	-	+						
SH 7-4	*B. cereus s.l.*	KENYA	+	+	-	+		0.00	884	IV	CC879-884	Kaminska *et al.* (2015)
SH 7-5	*B. cereus s.l.*	KENYA	+	+	-	+						
SH 7-6	*B. cereus s.l.*	KENYA	+	+	-	+						
SH 7-7	*B. cereus s.l.*	KENYA	+	+	-	+						
SH 7-8	*B. cereus s.l.*	KENYA	+	+	-	+						
SH 7-9	*B. cereus s.l.*	KENYA	+	+	-	+						
SH 7-10	*B. cereus s.l.*	KENYA	+	+	-	+						
SH 8-1	*B. cereus s.l.*	KENYA	+	+	-	+	MH618466	0.93	827	IV	S	Kaminska *et al.* (2015)
SH 8-2	*B. cereus s.l.*	KENYA	+	+	-	+						
SH 8-3	*B. cereus s.l.*	KENYA	+	+	-	+						
SH 8-4	*B. cereus s.l.*	KENYA	+	+	-	+						
SH 8-5	*B. cereus s.l.*	KENYA	+	+	-	+						
SH 8-6	*B. cereus s.l.*	KENYA	+	+	-	+						
SH 8-7	*B. cereus s.l.*	KENYA	-	+	-	-			1567	VIII	CC1566-1567	this study
SH 8-8	*B. cereus s.l.*	KENYA	+	+	-	+	MH618467	0.78	885	IV	S	Kaminska *et al.* (2015)
SH 8-9	*B. cereus s.l.*	KENYA	+	+	-	+						
SH 8-10	*B. cereus s.l.*	KENYA	+	+	-	+						
SH 8-8/12	*B. cereus s.l.*	KENYA	+	+	-	+						
SH 9-1	*B. cereus s.l.*	KENYA	+	+	-	+	MH618468	0.01	828	IV	S	Kaminska *et al.* (2015)
SH9-2	*B. cereus s.l.*	KENYA	+	+	-	-						
SH 9-3	*B. cereus s.l.*	KENYA	+	+	-	+						
SH 9-4	*B. cereus s.l.*	KENYA	-	+	-	+						
SH 9-5	*B. cereus s.l.*	KENYA	-	+	-	-						
SH 9-6	*B. cereus s.l.*	KENYA	+	+	-	+						
SH 9-7	*B. cereus s.l.*	KENYA	+	+	-	+						
SH 9-8	*B. cereus s.l.*	KENYA	+	+	-	+						
SH 9-9	*B. cereus s.l.*	KENYA	+	+	-	+						
SH 9-10	*B. cereus s.l.*	KENYA	+	+	-	+						
SH 10-1	*B. cereus s.l.*	KENYA	-	+	-	+						
SH 10-2	*B. cereus s.l.*	KENYA	+	+	-	-			829	V	S	Kaminska *et al.* (2015)
SH 10-3	*B. cereus s.l.*	KENYA	+	+	-	+						
SH 10-4	*B. cereus s.l.*	KENYA	+	+	-	+						
SH 10-5	*B. cereus s.l.*	KENYA	+	+	-	+						
SH 10-6	*B. cereus s.l.*	KENYA	+	+	-	+						
SH 10-7	*B. cereus s.l.*	KENYA	+	+	-	-			886	V	S	Kaminska *et al.* (2015)
SH 10-8	*B. cereus s.l.*	KENYA	+	+	-	+						
SH 10-9	*B. cereus s.l.*	KENYA	-	-	-	+						
SH 10-10	*B. cereus s.l.*	KENYA	+	+	-	+						
SH 10-11	*B. cereus s.l.*	KENYA	+	+	-	+						
BPN 02/2	*B. thuringiensis*	POLAND	-	+	-	-			709	VI	S	Drewnowska & Swiecicka (2013)
BPN 03/1	*B. thuringiensis*	POLAND	-	+	-	-			708	VI	S	Drewnowska & Swiecicka (2013)
BPN 05/2	*B. thuringiensis*	POLAND	+	+	-	-			675	VI	CC1507	Drewnowska & Swiecicka (2013)
BPN 05/3	*B. thuringiensis*	POLAND	+	+	-	-			724	VI	S	Drewnowska & Swiecicka (2013)
BPN 05/4	*B. thuringiensis*	POLAND	-	+	-	-			658	VI	CC1507	Drewnowska & Swiecicka (2013)
BPN 06/1	*B. thuringiensis*	POLAND	-	+	-	-			648	VI	S	Drewnowska & Swiecicka (2013)
BPN 07/3	*B. thuringiensis*	POLAND	+	+	-	-			659	VI	CC1507	Drewnowska & Swiecicka (2013)
BPN 08/1	*B. weihenstephanensis*	POLAND	+	+	-	-			649	VI	CC410-650	Drewnowska & Swiecicka (2013)
BPN 08/4	*B. thuringiensis*	POLAND	+	+	-	-			649	VI	CC410-650	Drewnowska & Swiecicka (2013)
BPN 10/2	*B. thuringiensis*	POLAND	+	+	-	-			660	VI	CC1507	Drewnowska & Swiecicka (2013)
BPN 12/1	*B. thuringiensis*	POLAND	-	+	-	-			737	VI	CC737-1519	Drewnowska & Swiecicka (2013)
BPN 13/1	*B. weihenstephanensis*	POLAND	-	+	-	-			650	VI	CC410-650	Drewnowska & Swiecicka (2013)
BPN 21/1	*B. weihenstephanensis*	POLAND	-	+	-	-			729	VI	CC729-742	Drewnowska & Swiecicka (2013)
BPN 23/2	*B. thuringiensis*	POLAND	+	+	-	-			676	VI	S	Drewnowska & Swiecicka (2013)
BPN 28/4	*B. weihenstephanensis*	POLAND	-	+	-	-			651	VI	S	Drewnowska & Swiecicka (2013)
BPN 29/1	*B. weihenstephanensis*	POLAND	-	+	-	-			678	VI	CC678	Drewnowska & Swiecicka (2013)
BPN 30/1	*B. weihenstephanensis*	POLAND	+	+	-	-			736	VI	S	Drewnowska & Swiecicka (2013)
BPN 30/3	*B. thuringiensis*	POLAND	-	+	-	-			657	VI	CC656-657	Drewnowska & Swiecicka (2013)

BPN 30/4	*B. thuringiensis*	POLAND	-	+	-	-		713	VI	S	Drewnowska & Swiecicka (2013)
BPN 32/3	*B. weihenstephanensis*	POLAND	-	+	-	-		652	VI	S	Drewnowska & Swiecicka (2013)
BPN 32/4	*B. thuringiensis*	POLAND	-	+	-	-		661	VI	S	Drewnowska & Swiecicka (2013)
BPN 33/4	*B. cereus s.l.*	POLAND	-	+	-	-		653	II	CC653-1508	Drewnowska & Swiecicka (2013)
BPN 34/4	*B. weihenstephanensis*	POLAND	-	+	-	-		737	VI	CC737-1519	Drewnowska & Swiecicka (2013)
BPN 35/2	*B. thuringiensis*	POLAND	-	+	-	-		679	VI	S	Drewnowska & Swiecicka (2013)
BPN 35/4	*B. weihenstephanensis*	POLAND	-	+	-	-		654	VI	S	Drewnowska & Swiecicka (2013)
BPN 36/2	*B. weihenstephanensis*	POLAND	+	+	-	-		650	VI	CC410-650	Drewnowska & Swiecicka (2013)
BPN 36/3	*B. weihenstephanensis*	POLAND	-	+	-	-		742	VI	CC729-742	Drewnowska & Swiecicka (2013)
BPN 36/4	*B. thuringiensis*	POLAND	-	+	-	-		710	VI	S	Drewnowska & Swiecicka (2013)
BPN 37/1	*B. weihenstephanensis*	POLAND	+	+	-	-		655	VI	CC410-650	Drewnowska & Swiecicka (2013)
BPN 37/2	*B. thuringiensis*	POLAND	+	+	-	-		662	VI	S	Drewnowska & Swiecicka (2013)
BPN 38/2	*B. weihenstephanensis*	POLAND	-	+	-	-		674	VI	S	Drewnowska & Swiecicka (2013)
BPN 38/4	*B. thuringiensis*	POLAND	-	+	-	-		743	VI	S	Drewnowska & Swiecicka (2013)
BPN 40/1	*B. thuringiensis*	POLAND	-	+	-	-		663	VI	S	Drewnowska & Swiecicka (2013)
BPN 40/2	*B. thuringiensis*	POLAND	-	+	-	-		710	VI	S	Drewnowska & Swiecicka (2013)
BPN 41/1	*B. thuringiensis*	POLAND	+	+	-	-		741	VI	S	Drewnowska & Swiecicka (2013)
BPN 42/1	*B. weihenstephanensis*	POLAND	+	+	-	-		730	VI	CC730-739	Drewnowska & Swiecicka (2013)
BPN 42/2	*B. thuringiensis*	POLAND	+	+	-	-		664	VI	S	Drewnowska & Swiecicka (2013)
BPN 43/2	*B. thuringiensis*	POLAND	-	+	-	-		680	VI	CC678	Drewnowska & Swiecicka (2013)
BPN 43/4	*B. weihenstephanensis*	POLAND	+	+	-	-		656	VI	CC656-657	Drewnowska & Swiecicka (2013)
BPN 44/2	*B. thuringiensis*	POLAND	+	+	-	-		665	V	CC218-223	Drewnowska & Swiecicka (2013)
BPN 45/4	*B. thuringiensis*	POLAND	+	+	-	-		665	V	CC218-223	Drewnowska & Swiecicka (2013)
BPN 47/1	*B. thuringiensis*	POLAND	-	+	-	-		666	VI	CC678	Drewnowska & Swiecicka (2013)
BPN 50/1	*B. thuringiensis*	POLAND	+	+	-	-		725	VI	S	Drewnowska & Swiecicka (2013)
BPN 50/3	*B. weihenstephanensis*	POLAND	-	+	-	-		657	VI	CC656-657	Drewnowska & Swiecicka (2013)
BPN 51/1	*B. thuringiensis*	POLAND	-	+	-	-		667	VI	S	Drewnowska & Swiecicka (2013)
BPN 51/2	*B. thuringiensis*	POLAND	-	+	-	-		677	VI	S	Drewnowska & Swiecicka (2013)
BPN 52/2	*B. weihenstephanensis*	POLAND	-	+	-	-		657	VI	CC656-657	Drewnowska & Swiecicka (2013)
BPN 53/1	*B. weihenstephanensis*	POLAND	-	+	-	-		692	VI	S	Drewnowska & Swiecicka (2013)
BPN 53/3	*B. thuringiensis*	POLAND	-	+	-	-		693	VI	S	Drewnowska & Swiecicka (2013)
BPN 54/1	*B. weihenstephanensis*	POLAND	-	+	-	-		731	VI	S	Drewnowska & Swiecicka (2013)
BPN 54/2	*B. thuringiensis*	POLAND	+	+	-	-		668	VI	CC1507	Drewnowska & Swiecicka (2013)
BPN 54/4	*B. thuringiensis*	POLAND	+	+	-	-		678	VI	CC678	Drewnowska & Swiecicka (2013)
BPN 55/2	*B. thuringiensis*	POLAND	-	+	-	-		669	VI	CC1507	Drewnowska & Swiecicka (2013)
BPN 55/4	*B. weihenstephanensis*	POLAND	+	+	-	-		656	VI	CC656-657	Drewnowska & Swiecicka (2013)
BPN 56/1	*B. thuringiensis*	POLAND	+	+	-	-		659	VI	CC1507	Drewnowska & Swiecicka (2013)
BPN 57/2	*B. thuringiensis*	POLAND	-	+	-	-		739	VI	CC730-739	Drewnowska & Swiecicka (2013)
BPN 57/3	*B. weihenstephanensis*	POLAND	-	+	-	-		738	VI	S	Drewnowska & Swiecicka (2013)
BPN 57/4	*B. thuringiensis*	POLAND	-	+	-	-		740	VI	S	Drewnowska & Swiecicka (2013)
BPN 58/4	*B. weihenstephanensis*	POLAND	-	+	-	-		708	VI	S	Drewnowska & Swiecicka (2013)
BPN 59/2	*B. thuringiensis*	POLAND	-	-	-	-		732	VI	CC732	Drewnowska & Swiecicka (2013)
BPN 60/1	*B. weihenstephanensis*	POLAND	+	+	-	-		624	VI	CC410-650	Drewnowska & Swiecicka (2013)
BPN 60/3	*B. thuringiensis*	POLAND	+	+	-	-		658	VI	CC1507	Drewnowska & Swiecicka (2013)
BPN023	*B. cereus s.l.*	POLAND	-	+	-	-					
BPN042	*B. cereus s.l.*	POLAND	-	+	-	-					
BPN063	*B. cereus s.l.*	POLAND	-	+	-	-					
BPN083	*B. cereus s.l.*	POLAND	-	+	-	-					
BPN093	*B. cereus s.l.*	POLAND	+	+	-	-					
BPN102	*B. weihenstephanensis*	POLAND	+	+	-	-		1507	VI	CC1507	this study
BPN103	*B. cereus s.l.*	POLAND	+	+	-	-					
BPN111	*B. cereus s.l.*	POLAND	+	+	-	-					
BPN114	*B. cereus s.l.*	POLAND	+	+	-	-					
BPN121	*B. weihenstephanensis*	POLAND	-	+	-	-		1519	VI	CC737-1519	this study
BPN124	*B. cereus s.l.*	POLAND	+	+	-	-					
BPN141	*B. cereus s.l.*	POLAND	+	+	-	-					
BPN143	*B. cereus s.l.*	POLAND	+	+	-	-					
BPN152	*B. cereus s.l.*	POLAND	-	+	-	-					
BPN154	*B. cereus s.l.*	POLAND	-	+	-	-					
BPN163	*B. cereus s.l.*	POLAND	-	+	-	-					
BPN164	*B. cereus s.l.*	POLAND	-	+	-	-					
BPN171	*B. cereus s.l.*	POLAND	-	+	-	-					
BPN181	*B. cereus s.l.*	POLAND	-	+	-	-					
BPN183	*B. cereus s.l.*	POLAND	-	+	-	-					
BPN191	*B. cereus s.l.*	POLAND	+	+	-	-					
BPN201	*B. cereus s.l.*	POLAND	+	+	-	-					
BPN211	*B. cereus s.l.*	POLAND	-	+	-	-		742	VI	CC729-742	this study
BPN215	*B. cereus s.l.*	POLAND	+	+	-	-					
BPN223	*B. cereus s.l.*	POLAND	+	+	-	-					
BPN242	*B. cereus s.l.*	POLAND	-	+	-	-					
BPN254	*B. cereus s.l.*	POLAND	-	+	-	-					
BPN263	*B. cereus s.l.*	POLAND	-	+	-	-					
BPN273	*B. cereus s.l.*	POLAND	-	+	-	-					
BPN292	*B. cereus s.l.*	POLAND	-	+	-	-					
BPN293	*B. cereus s.l.*	POLAND	+	+	-	-					
BPN312	*B. cereus s.l.*	POLAND	+	+	-	-					
BPN334	*B. cereus s.l.*	POLAND	+	+	-	-		1508	II	CC653-1508	this study
BPN391	*B. cereus s.l.*	POLAND	-	+	-	-					
BPN401	*B. mycoides*	POLAND	+	+	-	-		1568	VI	S	this study
BPN404	*B. cereus s.l.*	POLAND	+	+	-	-					
BPN 432	*B. weihenstephanensis*	POLAND	-	+	-	-		678	VI	CC678	this study
BPN461	*B. cereus s.l.*	POLAND	+	+	-	-					
BPN482	*B. cereus s.l.*	POLAND	+	+	-	-					
BPN491	*B. cereus s.l.*	POLAND	+	+	-	-					
BPN532	*B. cereus s.l.*	POLAND	-	+	-	-					
BPN 573	*B. weihenstephanensis*	POLAND	-	+	-	-		742	VI	CC729-742	this study
BPN601	*B. weihenstephanensis*	POLAND	-	+	-	-		1519	VI	CC737-1519	this study
BB 01/1	*B. cereus s.l.*	POLAND	+	+	-	-		722	II	S	Drewnowska & Swiecicka (2013)
BB 01/2	*B. cereus s.l.*	POLAND	+	+	-	-		705	V	CC705-723	Drewnowska & Swiecicka (2013)
BB 01/3	*B. cereus s.l.*	POLAND	+	-	-	-		295	II	CC1510	Drewnowska & Swiecicka (2013)
BB 02/1	*B. cereus s.l.*	POLAND	-	+	-	-		687	V	S	Drewnowska & Swiecicka (2013)
BB 02/3	*B. cereus s.l.*	POLAND	+	+	-	-		714	VI	CC714	Drewnowska & Swiecicka (2013)
BB 04/1	*B. cereus s.l.*	POLAND	+	+	-	-		644	II	S	Drewnowska & Swiecicka (2013)
BB 05/3	*B. cereus s.l.*	POLAND	+	+	-	-		688	VI	S	Drewnowska & Swiecicka (2013)
BB 06/1	*B. thuringiensis*	POLAND	+	+	-	-		633	V	S	Drewnowska & Swiecicka (2013)
BB 07/2	*B. thuringiensis*	POLAND	-	+	-	-		196	VI	CC196-447	Drewnowska & Swiecicka (2013)
BB 08/1	*B. cereus s.l.*	POLAND	+	+	-	+	0.60	630	II	CC1510	Drewnowska & Swiecicka (2013)
BB 10/5	*B. thuringiensis*	POLAND	+	+	-	-		636	V	CC218-223	Drewnowska & Swiecicka (2013)
BB 11/4	*B. cereus s.l.*	POLAND	-	-	-	-		699	VI	CC732	Drewnowska & Swiecicka (2013)
BB 12/1	*B. cereus s.l.*	POLAND	-	-	-	-		702	VI	S	Drewnowska & Swiecicka (2013)
BB 14/1	*B. weihenstephanensis*	POLAND	+	+	-	-		703	VI	CC714	Drewnowska & Swiecicka (2013)
BB 14/5	*B. cereus s.l.*	POLAND	-	-	-	-		699	VI	CC732	Drewnowska & Swiecicka (2013)
BB 15/1	*B. thuringiensis*	POLAND	-	+	-	-		196	VI	CC196-447	Drewnowska & Swiecicka (2013)
BB 15/2	*B. thuringiensis*	POLAND	+	+	-	-		637	V	S	Drewnowska & Swiecicka (2013)
BB 16/1	*B. cereus s.l.*	POLAND	-	-	-	-		638	II	CC638-1509	Drewnowska & Swiecicka (2013)

BB 16/2	*B. cereus s.l.*	POLAND	+	+	-	-			723	V	CC705-723	Drewnowska & Swiecicka (2013)
BB 17/4	*B. thuringiensis*	POLAND	+	+	-	-			218	V	CC218-223	Drewnowska & Swiecicka (2013)
BB 17/5	*B. cereus s.l.*	POLAND	-	+	-	-			631	V	S	Drewnowska & Swiecicka (2013)
BB 18/1	*B. cereus s.l.*	POLAND	+	+	-	-			733	II	CC564-733	Drewnowska & Swiecicka (2013)
BB 18/3	*B. cereus s.l.*	POLAND	-	+	-	-			700	II	S	Drewnowska & Swiecicka (2013)
BB 19/4	*B. cereus s.l.*	POLAND	-	+	-	-			632	II	CC551-632	Drewnowska & Swiecicka (2013)
BB 20/1	*B. weihenstephanensis*	POLAND	-	+	-	-			410	VI	CC410-650	Drewnowska & Swiecicka (2013)
BB 20/4	*B. cereus s.l.*	POLAND	+	+	-	-			686	II	CC1510	Drewnowska & Swiecicka (2013)
BB 21/3	*B. thuringiensis*	POLAND	-	+	-	-			218	V	CC218-223	Drewnowska & Swiecicka (2013)
BB 22/2	*B. weihenstephanensis*	POLAND	-	+	-	-			339	VI	S	Drewnowska & Swiecicka (2013)
BB 23/3	*B. weihenstephanensis*	POLAND	+	+	-	-			639	VI	S	Drewnowska & Swiecicka (2013)
BB 23/4	*B. cereus s.l.*	POLAND	+	+	-	-			701	V	CC218-223	Drewnowska & Swiecicka (2013)
BB 24/2	*B. thuringiensis*	POLAND	+	+	-	-			196	VI	CC196-447	Drewnowska & Swiecicka (2013)
BB 25/1	*B. cereus s.l.*	POLAND	-	+	-	-			551	II	CC551-632	Drewnowska & Swiecicka (2013)
BB 25/4	*B. cereus s.l.*	POLAND	+	+	-	-			689	II	S	Drewnowska & Swiecicka (2013)
BB 25/5	*B. cereus s.l.*	POLAND	+	+	-	-			705	V	CC705-723	Drewnowska & Swiecicka (2013)
BB 26/4	*B. cereus s.l.*	POLAND	-	+	-	-			564	II	CC564-733	Drewnowska & Swiecicka (2013)
BB 27/1	*B. thuringiensis*	POLAND	+	+	-	-			633	V	S	Drewnowska & Swiecicka (2013)
BB 27/4	*B. thuringiensis*	POLAND	-	+	-	-			715	VI	CC695	Drewnowska & Swiecicka (2013)
BB 28/4	*B. cereus s.l.*	POLAND	+	+	-	-			634	V	CC218-223	Drewnowska & Swiecicka (2013)
BB 29/2	*B. weihenstephanensis*	POLAND	+	+	-	-			635	VI	CC714	Drewnowska & Swiecicka (2013)
BB 30/5	*B. thuringiensis*	POLAND	+	+	-	-			704	VI	CC732	Drewnowska & Swiecicka (2013)
BB 31/5	*B. cereus s.l.*	POLAND	-	+	-	-			640	V	S	Drewnowska & Swiecicka (2013)
BB 32/5	*B. thuringiensis*	POLAND	-	+	-	-			705	V	CC705-723	Drewnowska & Swiecicka (2013)
BB 33/2	*B. weihenstephanensis*	POLAND	-	-	-	-			409	VI	S	Drewnowska & Swiecicka (2013)
BB 34/5	*B. cereus s.l.*	POLAND	+	+	-	-			705	V	CC705-723	Drewnowska & Swiecicka (2013)
BB 40/3	*B. thuringiensis*	POLAND	-	+	-	-			641	VI	S	Drewnowska & Swiecicka (2013)
BB 42/3	*B. thuringiensis*	POLAND	+	+	-	-			505	V	CC218-223	Drewnowska & Swiecicka (2013)
BB 43/5	*B. thuringiensis*	POLAND	+	+	-	-			642	V	CC218-223	Drewnowska & Swiecicka (2013)
BB 44/5	*B. thuringiensis*	POLAND	+	+	-	-			218	V	CC218-223	Drewnowska & Swiecicka (2013)
BB 45/2	*B. cereus s.s.*	POLAND	-	-	-	+	MH618455	1.05	643	IV	S	Drewnowska & Swiecicka (2013)
BB 46/2	*B. thuringiensis*	POLAND	-	+	-	-			715	VI	CC695	Drewnowska & Swiecicka (2013)
BB 47/4	*B. thuringiensis*	POLAND	+	+	-	-			218	V	CC218-223	Drewnowska & Swiecicka (2013)
BB 48/1	*B. thuringiensis*	POLAND	+	+	-	-			721	VI	S	Drewnowska & Swiecicka (2013)
BB 48/5	*B. thuringiensis*	POLAND	+	+	-	-			718	V	S	Drewnowska & Swiecicka (2013)
BB 50/5	*B. cereus s.l.*	POLAND	-	+	-	-			632	II	CC551-632	Drewnowska & Swiecicka (2013)
BB 51/3	*B. thuringiensis*	POLAND	-	+	-	-			223	V	CC218-223	Drewnowska & Swiecicka (2013)
BB 51/4	*B. cereus s.l.*	POLAND	+	+	-	+	MH618456	0.99	644	II	S	Drewnowska & Swiecicka (2013)
BB 52/3	*B. weihenstephanensis*	POLAND	+	+	-	-			421	VI	S	Drewnowska & Swiecicka (2013)
BB 52/5	*B. thuringiensis*	POLAND	-	+	-	-			665	V	CC218-223	Drewnowska & Swiecicka (2013)
BB 53/3	*B. cereus s.l.*	POLAND	-	+	-	-			728	VI	S	Drewnowska & Swiecicka (2013)
BB 54/3	*B. cereus s.l.*	POLAND	-	+	-	+		0.60	295	II	CC1510	Drewnowska & Swiecicka (2013)
BB 54/4	*B. cereus s.l.*	POLAND	+	+	-	+		0.90	644	II	S	Drewnowska & Swiecicka (2013)
BB 56/2	*B. thuringiensis*	POLAND	+	+	-	-			223	V	CC218-223	Drewnowska & Swiecicka (2013)
BB 56/3	*B. thuringiensis*	POLAND	+	+	-	+	MH618457	0.66	707	IV	S	Drewnowska & Swiecicka (2013)
BB 58/1	*B. thuringiensis*	POLAND	-	+	-	-			665	V	CC218-223	Drewnowska & Swiecicka (2013)
BB 58/3	*B. thuringiensis*	POLAND	-	+	-	-			196	VI	CC196-447	Drewnowska & Swiecicka (2013)
BB024	*B. cereus s.l.*	POLAND	+	+	-	-						
BB035	*B. cereus s.l.*	POLAND	+	+	-	-						
BB045	*B. cereus s.l.*	POLAND	-	+	-	-						
BB051	*B. cereus s.l.*	POLAND	+	+	-	-						
BB062	*B. cereus s.l.*	POLAND	-	+	-	+			1509	II	CC638-1509	this study
BB064	*B. cereus s.l.*	POLAND	-	+	-	-						
BB081	*B. cereus s.l.*	POLAND	+	+	-	+	MH618458	0.61	682	II	CC614	this study
BB093	*B. cereus s.l.*	POLAND	+	+	-	+	MH618459	0.00	1510	II	CC1510	this study
BB102	*B. cereus s.l.*	POLAND	-	+	-	-						
BB103	*B. cereus s.l.*	POLAND	+	+	-	-						
BB125	*B. cereus s.l.*	POLAND	-	+	-	-						
BB132	*B. cereus s.l.*	POLAND	-	+	-	-						
BB134	*B. cereus s.l.*	POLAND	-	+	-	-						
BB212	*B. cereus s.l.*	POLAND	+	+	-	-						
BB244	*B. cereus s.l.*	POLAND	+	+	-	-						
BB255	*B. cereus s.l.*	POLAND	+	+	-	-			312	II	S	this study
BB281	*B. cereus s.l.*	POLAND	+	+	-	+	MH618460	0.56	295	II	CC1510	this study
BB283	*B. cereus s.l.*	POLAND	+	+	-	-						
BB304	*B. cereus s.l.*	POLAND	+	+	-	-						
BB311	*B. cereus s.l.*	POLAND	+	+	-	-						
BB313	*B. cereus s.l.*	POLAND	+	+	-	-						
BB322	*B. cereus s.l.*	POLAND	+	+	-	-						
BB352	*B. cereus s.l.*	POLAND	-	+	-	-						
BB362	*B. cereus s.l.*	POLAND	-	+	-	-						
BB394	*B. cereus s.l.*	POLAND	+	+	-	-						
BB465	*B. cereus s.l.*	POLAND	+	+	-	-						
BB495	*B. cereus s.l.*	POLAND	-	+	-	-						
BB604	*B. cereus s.l.*	POLAND	-	+	-	+						
JAS 01/4	*B. weihenstephanensis*	POLAND	+	+	-	-			681	VI	CC681-1511	Drewnowska & Swiecicka (2013)
JAS 03/3	*B. thuringiensis*	POLAND	+	+	-	-			562	V	CC218-223	Drewnowska & Swiecicka (2013)
JAS 04/4	*B. thuringiensis*	POLAND	+	+	-	-			487	V	S	Drewnowska & Swiecicka (2013)
JAS 05/4	*B. cereus s.l.*	POLAND	+	+	-	-			312	II	S	Drewnowska & Swiecicka (2013)
JAS 05/5	*B. thuringiensis*	POLAND	-	+	-	-			695	VI	CC695	Drewnowska & Swiecicka (2013)
JAS 06/1	*B. cereus s.l.*	POLAND	+	+	-	-			222	VI	CC410-650	Drewnowska & Swiecicka (2013)
JAS 06/3	*B. cereus s.l.*	POLAND	+	+	-	-			617	VI	S	Drewnowska & Swiecicka (2013)
JAS 07/5	*B. cereus s.l.*	POLAND	+	+	-	-			312	II	S	Drewnowska & Swiecicka (2013)
JAS 08/1	*B. cereus s.l.*	POLAND	+	+	-	+	MH618445	1.12	682	II	CC614	Drewnowska & Swiecicka (2013)
JAS 09/5	*B. thuringiensis*	POLAND	+	+	-	-			487	V	S	Drewnowska & Swiecicka (2013)
JAS 10/2	*B. thuringiensis*	POLAND	+	+	-	+	MH618446	0.00	694	III	CC1512	Drewnowska & Swiecicka (2013)
JAS 11/1	*B. cereus s.l.*	POLAND	-	+	-	-			682	II	CC614	Drewnowska & Swiecicka (2013)
JAS 12/5	*B. thuringiensis*	POLAND	+	+	-	-			618	VI	S	Drewnowska & Swiecicka (2013)
JAS 13/1	*B. thuringiensis*	POLAND	+	+	-	-			487	V	S	Drewnowska & Swiecicka (2013)
JAS 15/2	*B. weihenstephanensis*	POLAND	+	+	-	-			222	VI	CC410-650	Drewnowska & Swiecicka (2013)
JAS 19/1	*B. weihenstephanensis*	POLAND	+	+	-	-			566	VI	CC410-650	Drewnowska & Swiecicka (2013)
JAS 21/1	*B. weihenstephanensis*	POLAND	+	+	-	-			566	VI	CC410-650	Drewnowska & Swiecicka (2013)
JAS 21/4	*B. thuringiensis*	POLAND	-	+	-	-			695	VI	CC695	Drewnowska & Swiecicka (2013)
JAS 21/5	*B. weihenstephanensis*	POLAND	+	+	-	-			410	VI	CC410-650	Drewnowska & Swiecicka (2013)
JAS 22/1	*B. thuringiensis*	POLAND	+	+	-	-			218	V	CC218-223	Drewnowska & Swiecicka (2013)
JAS 23/1	*B. weihenstephanensis*	POLAND	+	+	-	-			727	VI	CC410-650	Drewnowska & Swiecicka (2013)
JAS 23/4	*B. cereus s.l.*	POLAND	+	+	-	+		0.60	682	II	CC614	Drewnowska & Swiecicka (2013)
JAS 24/2	*B. cereus s.s.*	POLAND	+	+	-	+	MH618447	1.11	612	IV	S	Drewnowska & Swiecicka (2013)
JAS 26/2	*B. weihenstephanensis*	POLAND	+	+	-	-			619	VI	CC410-650	Drewnowska & Swiecicka (2013)
JAS 28/2	*B. cereus s.l.*	POLAND	+	+	-	-			613	II	S	Drewnowska & Swiecicka (2013)
JAS 30/2	*B. thuringiensis*	POLAND	-	+	-	-			696	VI	S	Drewnowska & Swiecicka (2013)
JAS 30/3	*B. weihenstephanensis*	POLAND	+	+	-	-			735	VI	CC410-650	Drewnowska & Swiecicka (2013)
JAS 31/2	*B. cereus s.l.*	POLAND	+	+	-	-			614	II	CC614	Drewnowska & Swiecicka (2013)
JAS 32/2	*B. thuringiensis*	POLAND	-	-	-	-			696	VI	S	Drewnowska & Swiecicka (2013)
JAS 32/4	*B. thuringiensis*	POLAND	-	+	-	-			487	V	S	Drewnowska & Swiecicka (2013)

JAS 35/2	*B. cereus s.s.*	POLAND	+	+	-	+	MH618448	0.87	612	IV	S	Drewnowska & Swiecicka (2013)
JAS 36/3	*B. cereus s.l.*	POLAND	-	+	-	-			682	II	CC614	Drewnowska & Swiecicka (2013)
JAS 39/1	*B. cereus s.l.*	POLAND	+	+	-	-			697	VI	CC410-650	Drewnowska & Swiecicka (2013)
JAS 41/1	*B. thuringiensis*	POLAND	+	+	-	-			620	VI	S	Drewnowska & Swiecicka (2013)
JAS 42/3	*B. thuringiensis*	POLAND	+	+	-	-			621	V	S	Drewnowska & Swiecicka (2013)
JAS 58/5	*B. thuringiensis*	POLAND	+	+	-	-			618	VI	S	Drewnowska & Swiecicka (2013)
JAS 60/4	*B. thuringiensis*	POLAND	+	+	-	-			683	VI	S	Drewnowska & Swiecicka (2013)
JAS 62/4	*B. thuringiensis*	POLAND	+	+	-	-			618	VI	S	Drewnowska & Swiecicka (2013)
JAS 63/5	*B. thuringiensis*	POLAND	-	+	-	-			622	V	S	Drewnowska & Swiecicka (2013)
JAS 67/5	*B. cereus s.l.*	POLAND	-	+	-	-			614	II	CC614	Drewnowska & Swiecicka (2013)
JAS 68/2	*B. weihenstephanensis*	POLAND	+	+	-	-			727	VI	CC410-650	Drewnowska & Swiecicka (2013)
JAS 74/1	*B. weihenstephanensis*	POLAND	-	-	-	-			720	VI	S	Drewnowska & Swiecicka (2013)
JAS 74/3	*B. weihenstephanensis*	POLAND	+	+	-	-			617	VI	S	Drewnowska & Swiecicka (2013)
JAS 76/3	*B. weihenstephanensis*	POLAND	+	+	-	-			698	VI	CC410-650	Drewnowska & Swiecicka (2013)
JAS 78/1	*B. thuringiensis*	POLAND	-	+	-	+	MH618449	0.00	695	VI	CC695	Drewnowska & Swiecicka (2013)
JAS 78/2	*B. weihenstephanensis*	POLAND	+	+	-	-			734	VI	CC410-650	Drewnowska & Swiecicka (2013)
JAS 80/5	*B. weihenstephanensis*	POLAND	+	+	-	-			683	VI	S	Drewnowska & Swiecicka (2013)
JAS 81/4	*B. weihenstephanensis*	POLAND	+	+	-	-			697	VI	CC410-650	Drewnowska & Swiecicka (2013)
JAS 82/3	*B. weihenstephanensis*	POLAND	+	+	-	-			719	VI	CC719-1514	Drewnowska & Swiecicka (2013)
JAS 83/2	*B. thuringiensis*	POLAND	+	+	-	+		0.00	683	VI	S	Drewnowska & Swiecicka (2013)
JAS 83/3	*B. weihenstephanensis*	POLAND	+	+	-	-			615	VI	CC410-650	Drewnowska & Swiecicka (2013)
JAS 84/1	*B. weihenstephanensis*	POLAND	-	+	-	-			684	VI	S	Drewnowska & Swiecicka (2013)
JAS 84/4	*B. weihenstephanensis*	POLAND	+	+	-	-			650	VI	CC410-650	Drewnowska & Swiecicka (2013)
JAS 85/1	*B. weihenstephanensis*	POLAND	+	+	-	-			410	VI	CC410-650	Drewnowska & Swiecicka (2013)
JAS 86/1	*B. weihenstephanensis*	POLAND	-	+	-	-			697	VI	CC410-650	Drewnowska & Swiecicka (2013)
JAS 90/2	*B. cereus s.l.*	POLAND	-	+	-	-			294	II	CC294	Drewnowska & Swiecicka (2013)
JAS 92/2	*B. cereus s.l.*	POLAND	-	-	-	-			616	II	S	Drewnowska & Swiecicka (2013)
JAS 94/5	*B. weihenstephanensis*	POLAND	+	+	-	-			726	VI	S	Drewnowska & Swiecicka (2013)
JAS 98/4	*B. thuringiensis*	POLAND	-	+	-	-			196	VI	CC196-447	Drewnowska & Swiecicka (2013)
JAS 100/3	*B. thuringiensis*	POLAND	-	+	-	-			562	V	CC218-223	Drewnowska & Swiecicka (2013)
JAS 100/4	*B. thuringiensis*	POLAND	+	+	-	-			717	VI	CC695	Drewnowska & Swiecicka (2013)
JAS014	*B. weihenstephanensis*	POLAND	+	+	-	-			1511	VI	CC681-1511	this study
JAS102	*B. cereus s.l.*	POLAND	+	+	-	+	MH618450	0.00	1512	III	CC1512	this study
JAS113	*B. cereus s.l.*	POLAND	-	+	-	-						
JAS122	*B. cereus s.l.*	POLAND	-	-	-	-						
JAS163	*B. cereus s.l.*	POLAND	-	+	-	-						
JAS181	*B. cereus s.l.*	POLAND	+	+	-	-						
JAS225	*B. cereus s.l.*	POLAND	-	+	-	-						
JAS234	*B. cereus s.l.*	POLAND	-	+	-	+	MH618451	0.00	1513	III	CC1512	this study
JAS253	*B. cereus s.l.*	POLAND	-	+	-	-						
JAS254	*B. cereus s.l.*	POLAND	-	+	-	-						
JAS291	*B. cereus s.l.*	POLAND	-	+	-	-						
JAS295	*B. cereus s.l.*	POLAND	-	+	-	-						
JAS334	*B. cereus s.l.*	POLAND	-	+	-	-						
JAS374	*B. cereus s.l.*	POLAND	-	+	-	-						
JAS382	*B. cereus s.l.*	POLAND	-	+	-	-						
JAS411	*B. cereus s.l.*	POLAND	+	+	-	-			487	V	S	this study
JAS413	*B. cereus s.l.*	POLAND	-	+	-	-						
JAS414	*B. cereus s.l.*	POLAND	-	+	-	-						
JAS422	*B. cereus s.l.*	POLAND	-	+	-	-						
JAS452	*B. cereus s.l.*	POLAND	-	+	-	-						
JAS483	*B. cereus s.l.*	POLAND	-	+	-	-						
JAS484	*B. cereus s.l.*	POLAND	-	+	-	-						
JAS511	*B. cereus s.l.*	POLAND	-	+	-	-						
JAS584	*B. cereus s.l.*	POLAND	-	+	-	-						
JAS622	*B. cereus s.l.*	POLAND	-	+	-	-						
JAS633	*B. cereus s.l.*	POLAND	-	+	-	-						
JAS634	*B. cereus s.l.*	POLAND	-	+	-	-						
JAS635	*B. cereus s.l.*	POLAND	-	+	-	-			695	VI	CC695	this study
JAS663	*B. cereus s.l.*	POLAND	+	+	-	+	MH618452	1.23	1520	IV	S	this study
JAS702	*B. cereus s.l.*	POLAND	+	+	-	+	MH618453	2.00	927	IV	CC1494	this study
JAS712	*B. cereus s.l.*	POLAND	-	+	-	+	MH618454	0.01	312	II	S	this study
JAS773	*B. cereus s.l.*	POLAND	-	-	-	-						
JAS783	*B. cereus s.l.*	POLAND	-	+	-	-						
JAS791	*B. cereus s.l.*	POLAND	-	+	-	-						
JAS823	*B. weihenstephanensis*	POLAND	+	+	-	-			1514	III	CC719-1514	this study
JAS884	*B. cereus s.l.*	POLAND	-	+	-	-						
JAS892	*B. cereus s.l.*	POLAND	-	+	-	-						
JAS905	*B. cereus s.l.*	POLAND	-	+	-	-						
JAS924	*B. cereus s.l.*	POLAND	-	+	-	-						
JAS964	*B. cereus s.l.*	POLAND	+	+	-	-						
JAS1004	*B. cereus s.l.*	POLAND	-	+	-	-			695	VI	CC695	this study
HD1	*B. thuringiensis*	USA	+	+	-	+		0.92	10	IV	S	NZ_CP004870.1
HD73	*B. thuringiensis*	unknown	+	+	-	+		1.11	8	IV	S	NC_020238.1
ATCC10987	*B. cereus s.s.*	CANADA	-	+	-	+		1.00	32	III	S	AE017194.1
ATCC14579	*B. cereus s.s.*	USA	+	+	-	+		0.62	4	IV	CC4-864	NC_004722
BCT-7112	*B. toyonensis*	JAPAN	+	+	-	-			111	V	CC218-223	NC_022781
DSM 22905	*B. cytotoxicus*	FRANCE	-	-	+	-			930	VII	S	NC_009674
ATCC10792	*B. thuringiensis*	CANADA	+	+	-	-			10	IV	S	NZ_CM000753
ATCC4342	*B. cereus s.s.*	unknown	+	+	-	-			38	III	S	CM000721.1
WSBC10360	*B. pseudomycoides*	unknown	nd	nd	nd	nd			87	I	S	MLST database
DSM 12442	*B. pseudomycoides*	USA	+	-	-	-			1305	I	S	NZ_CM000745
ATCC6462	*B. mycoides*	unknown	nd	nd	nd	nd			116	VI	S	MLST database
FSL W8-0169	*B. wiedmanii*	USA	+	+	-	+			1081	II	S	LOBC00000000
ATCC14578	*B. anthracis*	UK	-	+	-	-			1	III	S	ABJC00000000
F4810/72	*B. cereus s.s.*	USA	nd	nd	nd	nd			26	III	S	MLST database
DSMZ11821	*B. weihenstephanensis*	GERMANY	+	-	-	-			447	VI	CC196-447	BAUY00000000 (Liu *et al.* 2017)
MN5	*B. paranthracis*	THE PACIFIC OCEAN	-	+	-	+			761	III	S	MACE00000000 (Liu et al. 2017)
EB422	*B. pacificus*	THE PACIFIC OCEAN	-	+	-	+			32	III	S	MACD00000000 (Liu et al. 2017)
N24	*B. tropicus*	THE SOUTH CHINA SEA	-	+	-	+			771	III	S	MACG00000000 (Liu et al. 2017)
N35-10-2	*B. albus*	THE SOUTH CHINA SEA	+	+	-	-			775	II	S	MAOE00000000 (Liu et al. 2017)
0711P9-1	*B. mobilis*	THE INDIAN OCEAN	-	+	-	-			784	II	S	MACF00000000 (Liu et al. 2017)
TD41	*B. luti*	THE PACIFIC OCEAN	-	+	-	-			764	VIII	CC764-825	MACI00000000 (Liu et al. 2017)
TD42	*B. proteolyticus*	THE PACIFIC OCEAN	+	+	-	-			765	VI	S	MACH00000000 (Liu et al. 2017)
4049	*B. nitratireducens*	THE PACIFIC OCEAN	+	+	-	-			769	VI	S	MAOC00000000 (Liu et al. 2017)
NH24A2	*B. paramycoides*	THE SOUTH CHINA SEA	+	+	-	-			780	VI	S	MAOI00000000

[a] *B. cereus s.l., Bacillus cereus sensu lato; B. cereus s.s., Bacillus cereus sensu stricto; B. weihenstephanensis* (according to the latest classification considered as *B. mycoides*)

[b] Expression level of *cytK-2*

[c] Sequence Type

[d] Clonal Complex

Table S2. Genetic background of the enterotoxic potential of *B. cereus s.l.* isolates

Strain	MLST Group	Presence of virulence genes with the expression level[a,b]													Cells viability (% of control)[c]		GenBank Acces. No.
		cytK-2	*nheA*	*nheB*	*nheC*	*hblA*	*hblC*	*hblD*	*hly*-II	*hly*-III	*plcA*	*plcB*	*smase*	*clO*	Caco-2	HeLa	
AY 18-3	II		$+^{0.90}$	$+^{1.13}$	+	$+^{0.65}$	+	$+^{4.24}$	$+^{0.67}$	+	$+^{1.10}$	$+^{1.30}$	+	$+^{-2.50}$	19 ± 0.001	28 ± 0.79	QKQR00000000
BPN334	II		$+^{1.15}$	$+^{1.11}$	+	$+^{1.40}$	+	$+^{2.61}$	$+^{1.25}$	+	$+^{0.70}$	$+^{0.73}$	+	$+^{1.11}$	59 ± 0.01	91 ± 3.85	QOZW00000000
AR 2-1	II	$+^{0.00}$	$+^{0.76}$	$+^{1.43}$	+	$+^{1.27}$	+	$+^{0.80}$	$+^{1.75}$	+	$+^{0.88}$	$+^{1.40}$	+	$+^{0.57}$	36 ± 0.01	80 ± 3.85	QORY00000000
AY 3-1	II	$+^{0.00}$	$+^{0.71}$	$+^{0.68}$	+	$+^{2.32}$		$+^{2.55}$	$+^{0.45}$	+	$+^{0.62}$	$+^{0.72}$	+	$+^{0.76}$	20 ± 0.004	21 ± 1.53	QORZ00000000
SH 7-1	II	$+^{0.00}$	$+^{1.68}$	$+^{1.01}$	+	$+^{0.58}$	+	$+^{0.56}$	$+^{0.28}$	+	$+^{0.36}$	$+^{0.36}$	+	$+^{0.31}$	30 ± 0.013	19 ± 0.87	QNUD00000000
BB 51/4	II	$+^{0.99}$	$+^{2.26}$	$+^{1.29}$	+	$+^{3.32}$	+	$+^{2.32}$		+	$+^{0.99}$	$+^{1.45}$	+	$+^{0.89}$	29 ± 0.009	18 ± 1.98	QOSC00000000
BB081	II	$+^{0.61}$	$+^{0.97}$	$+^{0.71}$	+	$+^{1.87}$	+	$+^{1.32}$		+	$+^{1.22}$	$+^{0.88}$	+	$+^{1.50}$	30 ± 0.002	22 ± 0.19	QOZX00000000
**Average**[c]	II	0.23±0.15	1.20±0.21	1.05±0.11		1.63±0.37		2.06±0.48	0.63±0.25		0.84±0.11	0.98±0.16		1.09±0.28	31.9±0.007	39.9±1.87	
AR 18-7	III	$+^{0.01}$	$+^{0.71}$	$+^{0.48}$	+				$+^{0.27}$	+	$+^{0.62}$	$+^{0.77}$	+	$+^{0.36}$	79 ± 0.007	74 ± 1.54	QPAL00000000
BF 9-10	III	$+^{0.01}$	$+^{1.55}$	$+^{0.71}$	+	$+^{0.85}$	+	$+^{1.38}$	$+^{0.69}$	+	$+^{1.30}$	$+^{0.83}$	+	$+^{1.20}$	55 ± 0.008	91 ± 3.85	QNUC00000000
AY 1-10	III	$+^{0.01}$	$+^{1.60}$	$+^{1.10}$	+	$+^{0.92}$	+	$+^{1.01}$	$+^{0.71}$	+	$+^{0.98}$	$+^{1.00}$	+	$+^{1.01}$	30 ± 0.003	21 ± 1.98	QPEM00000000
JAS102	III	$+^{0.00}$	$+^{0.69}$	$+^{0.48}$	+	$+^{0.28}$	+	$+^{0.70}$	$+^{0.42}$	+	$+^{0.62}$	$+^{0.39}$	+	$+^{0.56}$	78 ± 0.005	72 ± 0.96	QPEK00000000
**Average**[c]	III	0.01±0.00	1.14±0.23	0.69±0.23		0.51±0.22		0.77±0.31	0.52±0.15		0.88±0.14	0.75±0.12		0.77±0.20	60.5±0.006	64.5±2.08	
AR 4-2	IV	$+^{0.66}$	$+^{0.77}$	$+^{0.58}$	+	$+^{0.67}$	+	$+^{0.57}$	$+^{0.49}$	+	$+^{0.88}$	$+^{0.58}$	+	$+^{0.85}$	35 ± 0.005	19 ± 1.98	CP031062-CP031064
AR 8-1	IV	$+^{0.61}$	$+^{0.83}$	$+^{0.62}$	+	$+^{0.56}$	+	$+^{0.54}$	$+^{0.47}$	+	$+^{1.15}$	$+^{0.56}$	+	$+^{1.19}$	23 ± 0.001	16 ± 0.87	QNUB00000000
BF 2-3	IV	$+^{0.81}$	$+^{1.40}$	$+^{0.99}$	+	$+^{0.67}$	+	$+^{0.99}$		+	$+^{1.75}$	$+^{0.56}$	+	$+^{1.06}$	42 ± 0.008	35 ± 1.12	QOSA00000000
AY 2-1	IV	$+^{0.55}$	$+^{0.91}$	$+^{0.64}$	+	$+^{0.74}$	+	$+^{0.84}$	$+^{0.47}$	+	$+^{0.88}$	$+^{0.96}$	+	$+^{1.33}$	35 ± 0.005	21 ± 1.53	QPEL00000000
TE 8-1	IV	$+^{0.92}$	$+^{1.10}$	$+^{0.60}$	+	$+^{0.78}$	+	$+^{1.03}$		+	$+^{0.82}$	$+^{0.90}$	+	$+^{1.38}$	32 ± 0.002	24 ± 1.12	QPET00000000
SH 8-8	IV	$+^{0.78}$	$+^{1.02}$	$+^{0.67}$	+	$+^{1.40}$	+	$+^{0.76}$	$+^{0.47}$	+	$+^{0.91}$	$+^{0.86}$	+	$+^{1.18}$	36 ± 0.003	20 ± 1.98	CP031065-CP031067
BB 56/3	IV	$+^{0.66}$	$+^{0.87}$	$+^{1.10}$	+	$+^{0.46}$	+	$+^{0.38}$	$+^{0.69}$	+	$+^{0.83}$	$+^{0.68}$	+	$+^{0.81}$	96 ± 0.002	20 ± 2.51	QOSB00000000
JAS 24/2	IV	$+^{1.11}$	$+^{0.75}$	$+^{0.76}$	+	$+^{0.60}$	+	$+^{0.46}$		+	$+^{1.30}$	$+^{0.47}$	+	$+^{0.77}$	53 ± 0.007	25 ± 0.79	CP031068-CP031070

**Average**[c]	IV	0.76±0.06	0.96±0.08	0.75±0.07		0.74±0.10		0.70±0.09	0.35±0.10		1.07±0.11	0.70±0.07		1.07±0.08	44.0±0.004	22.5±1.49	
AR 13-1	V		$+^{1.01}$	$+^{0.56}$	+	$+^{2.33}$	+	$+^{1.58}$		+	$+^{0.71}$	$+^{0.61}$	+	$+^{1.34}$	92 ± 0.005	85 ± 3.85	QNUA00000000
BPN401	VI		$+^{1.16}$	$+^{0.37}$	+	$+^{1.43}$	+	$+^{0.91}$		+	$+^{1.07}$	$+^{0.84}$	+	$+^{0.99}$	97 ± 0.006	93 ± 3.85	CP031071-CP031077
SH 5-2	VIII		$+^{1.30}$	$+^{0.80}$	+					+		$+^{0.81}$	+	$+^{0.94}$	56 ± 0.009	57 ± 1.15	QOSD00000000
ATCC10987	III	$+^{1.00}$	$+^{0.79}$	$+^{1.57}$	+					+	$+^{0.77}$	$+^{0.94}$	+	$+^{0.73}$	42 ± 0.004	23 ± 2.51	NZ_CM000753
ATCC14579	IV	$+^{0.62}$	$+^{1.00}$	$+^{1.00}$	+	$+^{1.00}$	+	$+^{1.00}$	$+^{1.00}$	+	$+^{1.00}$	$+^{1.00}$	$+^{1.00}$	$+^{1.00}$	21 ± 0.01	27 ± 1.12	ABJC00000000

[a] Non-Hemolytic Enterotoxin (NHE) encoding genes (*nheA, nheB, nheC*); Haemolysin BL (HBL) genes (*hblA, hblC, hblD*); Hemolysin-II (*hly*-II); Hemolysin-III (*hly*-III); 1-phosphatidylinositol phosphodiesterase (*plcA);* Phospholipases C (*plcB*); Sphingomyelinase C (*smase*); thiol-activated cytolysin O (*clO*)

[b] Expression level of gene is shown in superscript

[c] ±SE

Table S3. PCR primers used in this study.

Target gene	Primer name	Nucleotide	Product size (bp)	Thermal profile	Reference
**PCR screening**					
nheA	nheA843A<br>nheA344S	5'-GTTTTTATTGCTTCATCGGCT-3'<br>5'-TACGCTAAGGAGGGGCA-3'	499	3 min, 95 °C → 30x (30 s, 95 °C → 30 s, 57 °C → 30 s, 72 °C) → 7min, 72 °C	[1]
hblA	HBLA1<br>HBLA2	5'-GTGCAGATGTTGATCCCGAT-3'<br>5'-ATGCCACTGCGTGGACATAT-3'	320	3 min, 95 °C → 30x (15 s, 95 °C → 30 s, 59 °C → 45 s, 72 °C) → 7min, 72 °C	[2]
cytK-1	CK1F<br>CK1R	5'-CAATTCCAGGGGCAAGTGTC-3'<br>5'-CCTCGTGCATCTGTTTCATGAG-3'	426	5 min, 94 °C → 30x (15 s, 94 °C → 30 s, 57 °C → 60 s, 72 °C) → 7min, 72 °C	[3]
cytK	CKF<br>CKR	5'-ACAGATATCGGKCAAAAYGC-3'<br>5'-CTTGACSWTTGACCCAACCT-3'	809	5 min, 94 °C → 30x (15 s, 94 °C → 30 s, 60 °C → 60 s, 72 °C) → 7min, 72 °C	[3]
cytK-2	CytKF2<br>CytKR2	5'-TGTAATACGACATATATCGACA-3'<br>5'-CRTCTTTTACGTTGTTC-3'	982	5 min, 94 °C → 30x (20 s, 94 °C → 30 s, 54 °C → 60 s, 72 °C) → 7min, 72 °C	this study
**qRT-PCR analysis**					
udp	udpF<br>udpR	5'-ACTAGAGAAACTTGGAAATGATCG-3'<br>5'-GACGCTTAATTGCACGGAAC-3'	101	2 min, 94 °C → 40x (30 s, 95 °C → 30 s, 58 °C → 30 s, 72 °C)	[4]
cytK-2	RT-cytK-F2<br>RT-cytK-R2	5'-GACAATGTCTTTAAAGGTAACT-3'<br>5'-CTTATAGTAGGATTTGCTTTTA-3'	104	2 min, 94 °C → 40x (20 s, 95 °C → 30 s, 56 °C → 20 s, 72 °C)	this study
hblA	hblA_F<br>hblA_R	5'-AATATTAATGAAATGCACAA-3'<br>5'-CCCGAATATTGAGAATCT-3'	95	2 min, 94 °C → 40x (20 s, 95 °C → 30 s, 54 °C → 20 s, 72 °C)	this study
hblD	hblD_F<br>hblD_R	5'-GCAAAAACAATGATTAAGCAAC-3'<br>5'TCGTAATAATTTTGAAATTGTTCATC-3'	206	2 min, 94 °C → 40x (20 s, 95 °C → 30 s, 56 °C → 20 s, 72 °C)	this study
nheA	nheA_F<br>nheA_R	5’-TTCCTGTAAGCGCTTACG-3'<br>5'-CTTCGGATTATATTCATCAATC-3'	251	2 min, 94 °C → 40x (20 s, 95 °C → 30 s, 58 °C → 20 s, 72 °C)	this study

nheB	nheB_F<br>nheB_R	5'-ATGGCAGTATTTGCAGCA-3'<br>5'-GCCTTCTGGTCCTAATGA-3'	135	2 min, 94 °C → 40x (20 s, 95 °C → 30 s, 58 °C → 20 s, 72 °C)	this study
hlyII	hlyII_F1<br>hlyII_R1	5'-AATACAATGACTTCAGCAAAGG-3'<br>5'-TAAGATACACTTTCTTTCCAAGCA-3'	132	2 min, 94 °C → 40x (20 s, 95 °C → 30 s, 58 °C → 20 s, 72 °C)	this study
clO	Clo_F<br>Clo_R	5'-TGATGTATCTTGGGATGAA-3'<br>5'-GTTGTTCCTCCAATTGAAAC-3'	259	2 min, 94 °C → 40x (20 s, 95 °C → 30 s, 56 °C → 20 s, 72 °C)	this study
plcA	plcA_F<br>plcA_R	5'-GCACGAATTTCAATTCCAG-3'<br>5'-TCTTGCGTCATTCCCCA-3'	85	2 min, 94 °C → 40x (20 s, 95 °C → 30 s, 58 °C → 20 s, 72 °C)	this study
plcB	plcB_F<br>plcB_R	5'-TATGAAAATCCTTATTATGATAATAG -3'<br>5’-TACAAAGTTTTCATATTTAGAATGG-3'	282	2 min, 94 °C → 40x (20 s, 95 °C → 30 s, 56 °C → 20 s, 72 °C)	this study
**MLST analysis**					www.pubmlst.org/bcereus/
glpF	glpF_F<br>glpF_R	5'-GCGTTTGTGCTGGTGTAAGT-3'<br>5'-CTGCAATCGGAAGGAAGAAG-3'	548	5 min, 94 °C → 30x (15 s, 94 °C → 30 s, 59 °C → 40 s, 72 °C) → 7min, 72 °C	
gmk	gmk_F<br>gmk_R	5'-ATTTAAGTGAGGAAGGGTAGG-3'<br>5'-GCAATGTTCACCAACCACAA-3'	599	5 min, 94 °C → 30x (15 s, 94 °C → 30 s, 56 °C → 40 s, 72 °C) → 7min, 72 °C	
ilvD	ilvD_F<br>ilvD_R	5'-CGGGGCAAACATTAAGAGAA-3'<br>5'-GGTTCTGGTCGTTTCCATTC-3'	553	5 min, 94 °C → 30x (15 s, 94 °C → 30 s, 58 °C → 40 s, 72 °C) → 7min, 72 °C	
	ilvD4_F<br>ilvD2_R	5'-GCAGAGATTAAAGATAAGGA-3'<br>5'-GTTACCATTTGTGATAACGC-3'	568	5 min, 94 °C → 30x (15 s, 94 °C → 30 s, 50 °C → 40 s, 72 °C) → 7min, 72 °C	
pta	pta_F<br>pta_R	5'-GCAGAGCGTTTAGCAAAAGAA-3'<br>5'-TGCAATGCGAGTTGCTTCTA-3'	575	5 min, 94 °C → 30x (15 s, 94 °C → 30 s, 56 °C → 40 s, 72 °C) → 7min, 72 °C	
pur	pur_F<br>pur_R	5'-CTGCTGCGAAAAATCACAAA-3'<br>5'-CTCACGATTCGCTGCAATAA-3'	534	5 min, 94 °C → 30x (15 s, 94 °C → 30 s, 56 °C → 40 s, 72 °C) → 7min, 72 °C	
pycA	pycA_F<br>pycA_R	5'-GCGTTAGGTGGAAACGAAAG-3'<br>5'-CGCGTCCAAGTTTATGGAAT-3'	549	5 min, 94 °C → 30x (15 s, 94 °C → 30 s, 57 °C → 40 s, 72 °C) → 7min, 72 °C	

tpi	tpi_F<br>tpi_R	5'-GCCCAGTAGCACTTAGCGAC-3'<br>5'-CCGAAACCGTCAAGAATGAT-3'	557	5 min, 94 °C → 30x (15 s, 94 °C → 30 s, 58 °C → 40 s, 72 °C) → 7min, 72 °C

[1] Ghelardi, E.; Celandroni, F.; Salvetti, S.; Barsotti, C.; Baggiani, A.; Senesi, S. Identification and characterization of toxigenic *Bacillus cereus* isolates responsible for two food-poisoning outbreaks. *FEMS Microbiol. Lett* . **2002**, *208* , 129-134.

[2] Hansen, B. M.; Hendriksen, N. B. Detection of enterotoxic Bacillus cereus and Bacillus thuringiensis strains by PCR analysis. Appl. Environ. Microbiol. 2001, 67, 185-189.

[3] Guinebretière, M.H.; Thompson, F.L.; Sorokin, A.; Normand, P.; Dawyndt, P.; Ehling-Schulz, M.; Svensson, B.; Sanchis, V.; Nguyen-The, C.; Heyndrickx, M.; De Vos, P. Ecological diversification in the Bacillus cereus group. Environ. Microbiol. 2008, 10, 851-865.

[4] Reiter, L.; Kolstø, A.B.; Piechler, A.P. Reference genes for quantitative, reverse-transcription PCR in Bacillus cereus group strains throughout the bacterial life cycle. J. Microbiol. Meth. 2011, 86, 210-217.
